# Nanopore sequencing for precise detection of *Mycobacterium tuberculosis* and drug resistance: a retrospective multicenter study in China

**DOI:** 10.1128/jcm.01813-24

**Published:** 2025-03-19

**Authors:** Shanshan Yu, Ning Liu, Zhouhua Xie, Yi Zeng, Hua Wang, Qian Wang, Peibo Li, Haoran Li, Jinhao Sun, Qingdong Zhu, Weiwei Gao, Hongcang Gu, Fuyou Liu, Peisong Xu, Yunfei Wang, Liang Li, Yu Pang

**Affiliations:** 1 Department of Bacteriology and Immunology, Beijing Chest Hospital, Capital Medical University 117550 , Beijing, China; 2 Beijing Tuberculosis and Thoracic Tumor Research institute, Beijing, China; 3 Hebei Chest Hospital, Hebei, China; 4 The Fourth People’s Hospital of Nanning, Nanning, China; 5 The Second Hospital of Nanjing 531909 , Nanjing, China; 6 Anhui Chest Hospital 552822 , Hefei, China; 7 Hangzhou Shengting Medical Technology Co., Ltd, Hangzhou, China; 8 Chongqing Public Health Medical Center 567508 , Chongqing, China; 9 Anhui Province Key Laboratory of Medical Physics and Technology, Institute of Health and Medical Technology, Hefei Institutes of Physical Science, Chinese Academy of Sciences 53040 , Hefei, China; University of Manitoba, Winnipeg, Manitoba, Canada

**Keywords:** tuberculosis, diagnosis, drug resistance, nanopore sequencing

## Abstract

Tuberculosis (TB) management in endemic regions often grapples with resource constraints, including the scarcity of *Mycobacterium tuberculosis* (*M.tb*) culture laboratories. The emergence of *M.tb* strains with complex drug resistance profiles necessitates rapid and comprehensive drug susceptibility testing (DST) to guide patient treatment. However, traditional phenotypic DST (pDST) for *M.tb* is costly and time-consuming. In this study, we retrospectively enrolled 829 participants from six specialized TB treatment hospitals in China from September 2022 to July 2023. The diagnostic performance of TBseq test, a targeted-nanopore sequencing assay, was compared head-to-head with *M.tb* culture, acid-fast bacillus smear, quantitative polymerase chain reaction, and Xpert MTB/RIF Ultra by using clinical diagnosis as the reference standard. Subsequently, pDST for seven anti-TB drugs (rifampicin, isoniazid, ethambutol, streptomycin, levofloxacin, amikacin, and capreomycin) was performed. The resistance predictions provided by the TBseq test were compared with pDST results, which were used as a reference standard. The performance estimates of TBseq test were quantified through sensitivity, specificity, positive predictive value, negative predictive value, and the area under the receiver operating characteristic curve (AUC), providing a comprehensive assessment of its diagnostic accuracy. We found that TBseq test demonstrated significantly superior diagnostic performance for TB compared to other methods, achieving a sensitivity of 90.9% (95% CI: 88.9%–93.0%), specificity of 93.0% (95% CI: 97.2%–99.5%), and an AUC of 0.92 (95% CI: 0.876–0.963). TBseq test also exhibited robust predictive capabilities for drug resistance to the seven anti-TB drugs, with sensitivity and specificity consistently above 90% for all drugs. The AUC values ranged from 0.919 to 0.998, indicative of high diagnostic accuracy in forecasting drug resistance.

**IMPORTANCE:**

Our results show that TBseq test offers excellent identification performance for tuberculosis (TB), significantly outperforming *Mycobacterium tuberculosis* (*M.tb)* culture, acid-fast bacillus (AFB) smear, qPCR, and Xpert MTB/RIF. Its diagnostic accuracy for anti-TB drug resistance is also superior, with sensitivity and specificity above 90% for all drugs tested. This method can be integrated into routine clinical diagnostic workflows, enabling early diagnosis and reporting of drug resistance simultaneously.

## INTRODUCTION

Tuberculosis (TB), an ancient infectious disease caused by *Mycobacterium tuberculosis* (*M.tb*), remains a significant threat to global health. The Global Tuberculosis Report 2024, compiled by the World Health Organization (WHO) reveals that TB probably returned to be the world’s leading cause of death from a single infectious agent in 2023, which was replaced by coronavirus disease before. In 2023, the global incidence of newly diagnosed TB cases reached 10.8 million, with an estimated 1.25 million deaths attributed to TB (1, 2).

Drug-resistant TB (DR-TB), characterized by resistance to first-line anti-TB drugs, further complicates treatment and poses a substantial public health challenge (3). The WHO classifies DR-TB into multiple classes, including multidrug-resistant TB (MDR-TB), extensively DR-TB (XDR-TB), and pre-extensively DR-TB (pre-XDR-TB). Traditionally, patients afflicted with drug-resistant strains endured lengthier and more intricate treatment regimens, often entailing cocktails of second-line drugs, leading to heightened adverse effects and substantial economic strain. However, the recent roll-out of the BPaL(M) regimen, which includes bedaquiline, pretomanid, and linezolid (with or without moxifloxacin), has simplified and shortened the treatment duration for many patients, potentially reducing adverse effects and economic burden. The BPaL(M) regimen, recommended by the WHO, is a shorter and more effective treatment option for MDR-TB and XDR-TB. This regimen has shown promising results in clinical trials, with high cure rates and fewer side effects compared to traditional second-line drug regimens (3, 4). Alarmingly, there were an estimated 450,000 new rifampicin (RIF)-resistant TB cases in 2021 globally, a 3% increment from 2020, marking the first rise since 2015. With only 161,000 of these patients receiving treatment, nearly two-thirds are not diagnosed or treated promptly, underscoring the pressing need for rapid drug resistance prediction to optimize antibiotic therapies and improve patient outcomes.

Current diagnostic protocols for DR-TB primarily rely on *in vitro* drug susceptibility testing (DST), encompassing phenotypic and molecular approaches (5). Phenotypic DST (pDST), although considered the gold standard, is time-consuming and susceptible to environmental influences (6). Conversely, molecular assays such as Xpert MTB/RIF, which harnesses real-time PCR, offer quicker results but are limited to detect prevalent mutations and lack comprehensive resistance profiles (7, 8). Second-generation sequencing technologies, with their speed and high throughput, have attempted to bridge this gap by identifying specific mutations, yet their adoption is hindered by sequence biases, high resource demands, and operational complexity (9, 10).

Third-generation sequencing technologies, exemplified by Pacific Biosciences’ Single-Molecule Real-Time sequencing and Oxford Nanopore Technologies’ nanopore sequencing, have garnered attention due to their extended read lengths (tens to hundreds of kb), enhanced throughput, expedited timelines, and simplified usage, making them increasingly viable for clinical application (11–15). In particular, nanopore sequencing eliminates the reliance on DNA polymerases and facilitates real-time analysis, demonstrating exceptional promise in TB diagnostics and resistance profiling (16, 17). Preliminary studies have reported near-perfect concordance with conventional methods, rapid turnaround times ranging from hours to days, and improved sensitivity and specificity over established tests, thereby highlighting the potential of this technology to revolutionize TB and DR-TB diagnosis (18–20).

Despite these advances, the application of nanopore sequencing in TB screening and DR-TB diagnosis using clinical specimens is not yet widespread. Large-scale clinical studies validating the applicability and reliability of nanopore sequencing for DR-TB are scarce. This study aims to fill this gap by evaluating the diagnostic performance of TBseq test, a targeted nanopore sequencing assay, in a large cross-sectional study involving 829 TB patients. The TBseq test (Hangzhou ShengTing Medical Technology Co., China) is a diagnostic tool based on targeted NGS that is used for the simultaneous identification of mycobacterial species and the prediction of drug resistance of MTBC strains. It is directly applicable to clinical specimens such as sputum and bronchoalveolar lavage fluid (BALF). According to the WHO operational handbook for TB-Module 3, 2024, TBseq test is endorsed for ethambutol (EMB) resistance detection. For other drugs, further improvements to the product and a review of the evidence will be necessary before clinical use can be recommended for those specific resistances (21).

Our comprehensive multicenter study is the first of its scale to systematically assess the performance of TBseq test in diagnosing TB and predicting drug resistance, focusing on specificity, sensitivity, positive predictive value (PPV), negative predictive value (NPV), and area under the receiver operating characteristic curve (AUC), as well as its cost and turnaround time. The insights gained from this research are expected to facilitate the adoption of rapid and accurate TB drug resistance detection assays, enabling timely clinical interventions and contributing to the global effort to reduce the TB disease burden.

## MATERIALS AND METHODS

### Study design and participant enrollment

We conducted a retrospective multicenter study from September 2022 to July 2023, enrolling 1,161 patients with symptoms suggestive of active TB at six specialized hospitals across China, including Beijing Chest Hospital, Hebei Provincial Chest Hospital, Anhui Provincial Chest Hospital, The Second Hospital of Nanjing, Chongqing Public Health Medical Center, and Nanning Fourth People’s Hospital. Eligible subjects underwent a comprehensive TB screening incorporating acid-fast bacillus (AFB) smear, *M.tb* culture, pDST, GeneXpert MTB/RIF assay (Cepheid, Sunnyvale, USA), and TBseq test (Hangzhou ShengTing Medical Technology Co., China), following established methodologies (22,16). Clinical data were collected through a thorough review of patient medical records, including a detailed examination of inpatient and outpatient files, laboratory test results, radiological reports, and any other pertinent clinical documentation. Data abstraction was conducted by trained research staff who were blinded to the study’s objectives. The data collection process was standardized to ensure consistency and accuracy across all participants.

### Participant selection

Patients presenting with TB-related clinical symptoms, such as cough, fever, and night sweats, together with radiological findings indicative of active TB, and with respiratory or non-respiratory samples (e.g., sputum, BALF, tissue, and pus) available for testing were enrolled in our analysis as suspected TB cases. Clinical specimens, including sputum, BALF, pleural effusion, ascites urine, cerebrospinal fluid (CSF), and pus, were subjected to the aforementioned tests.

The exclusion criteria were as follows: (i) patients without complete case data, (ii) patients missing nanopore sequencing results, and (iii) patients with co-infections of other organs or system. Following exclusions, 829 out of 1,161 patients were included in the subsequent data analysis. Missing samples include those that were never tested by TBseq or those that were tested but resulted in technical failures or partial results that did not meet quality control standards. If an initial TBseq attempt was unsuccessful, we attempted retesting when feasible. Of the 332 excluded patients, 67 (20.2%) were excluded solely because they had no TBseq result. The remaining 265 patients (79.8%) were excluded for other reasons, such as missing clinical data or co-infection of other organs or system. The patient distribution across the participating institutions was as follows: 334 patients from Hebei Chest Hospital, 186 patients from the Fourth People’s Hospital of Nanning, 162 patients from the Second Hospital of Nanjing, 55 patients from Beijing Chest Hospital, 50 patients from Anhui Chest Hospital, and 42 patients from Chongqing public health medical center.

### Clinical diagnostic criteria

The clinical diagnostic criteria for TB in this study were based on a combination of clinical symptoms, imaging findings, and laboratory results. The specific criteria used were as follows. (i) Clinical symptoms: persistent cough (≥3 weeks), fever, night sweats, unintentional weight loss, and hemoptysis. (ii) Imaging findings: chest X-ray or CT scan showing abnormalities consistent with TB, such as infiltrates, cavitations, or pleural effusions. (iii) Laboratory results: positive AFB smear or culture from any clinical sample, positive Xpert MTB/RIF Ultra results; elevated erythrocyte sedimentation rate or C-reactive protein levels. (iv) Histopathological examination: presence of granulomas or caseating necrosis in tissue biopsies. (v) Response to treatment: improvement in clinical symptoms and imaging findings after initiation of anti-TB treatment. A diagnosis of active TB was confirmed if the patient met at least two of the above criteria, with at least one positive laboratory result (AFB smear, culture, or Xpert MTB/RIF Ultra). For suspected extrapulmonary TB, a positive histopathological examination or response to treatment was also considered confirmatory.

### Sample collection

In this study, clinical samples from patients were collected in various forms, including sputum, BALF, tissue, pus, CSF, urine, pleural effusion, and ascites. The choice of sample type was based on the site of disease and practical clinical considerations, as follows.

#### Sputum sample collection

Sputum samples are the standard, minimally invasive method for diagnosing TB. In this study, sputum samples were the primary specimen type. However, sputum collection depends on the patient’s ability to produce sputum, and the quality of sputum can vary. The frequency of sputum collection was determined based on the patient’s ability to produce sputum and the quality of the samples.

#### BALF sample collection

BALF sampling is often used when patients are unable to produce sputum or when sputum samples are insufficient for analysis. In our study, BALF was prioritized for patients who had difficulty producing sputum or when the treating physician deemed a more invasive diagnostic method necessary.

#### Tissue sample collection

Tissue samples were obtained from biopsy procedures, particularly in cases where extrapulmonary TB was suspected. These samples were collected from sites such as lymph nodes, pleura, or other affected organs. Tissue biopsies provide direct access to the infected tissue, offering a high diagnostic yield and allowing for histopathological examination and culture.

#### Pus sample collection

Pus samples were collected from abscesses or draining sinuses, typically in cases of extrapulmonary TB. The collection of pus samples was performed under sterile conditions to avoid contamination.

#### CSF sample collection

CSF samples were collected from patients suspected of having tuberculous meningitis. A lumbar puncture was performed to obtain CSF, which was then analyzed for the presence of mycobacterial DNA.

#### Urine sample collection

Urine samples were collected from patients with suspected genitourinary TB. Urine is a non-invasive sample type and can be easily obtained.

#### Pleural effusion and ascites sample collection

Pleural effusion samples were collected from patients with suspected pleural TB via thoracentesis under ultrasound guidance to ensure accuracy and safety. Ascites samples were obtained through paracentesis using a comparable ultrasound-guided technique.

Each sample type was selected based on the clinical presentation and the need for accurate diagnosis. The collection methods were standardized to ensure consistency and reliability of the results. All samples were processed and stored according to established protocols to maintain their integrity and suitability for downstream analyses.

The collected clinical samples were divided equally into five unbiased segments. Three segments were sent to the microbiology laboratory of the hospital for bacterial culture, AFB smear, and Xpert MTB/RIF Ultra analysis. The other two segments were immediately sent to Hangzhou ShengTing Medical Co. Ltd. for TNPseq and qPCR processing using cold chain logistics, with each segment undergoing DNA extraction using the same standardized methodology to ensure consistency across assays. Both laboratories followed the “National Clinical Testing Practice for Pathogen Detection in Clinical Microbiology Laboratory” protocol for standard analytical procedures.

### Conventional laboratory examinations

#### AFB smear

The AFB smear microscopy was conducted in accordance with the national TB program guidelines. Briefly, sputum specimens were spread on slides and processed as previously described, with body fluid samples centrifuged for smear preparation (16). Subsequent culture of *M.tb* was conducted on Lowenstein–Jensen (LJ) medium or liquid medium (MGIT 960 system) following the routine diagnostic procedures.

#### Xpert MTB/RIF Ultra Assay

For the Xpert MTB/RIF Ultra assay, the procedure was carried out according to the manufacturer’s instructions (23). Briefly, 1 mL of clinical specimen was mixed with the sample processing solution, incubated, and then transferred into the reaction cartridge for analysis. Results were interpreted directly from the detection system’s result window.

#### Phenotypic DST

The phenotypic drug susceptibility of *M.tb* isolates was determined using microplate dilution method as previously described (24). Briefly, the freshly grown *M.tb* isolates to be tested were suspended by adding a small loop of bacteria in distilled saline. After vigorous mixing for 1 min, a suspension of *M.tb* isolate was adjusted to the density of a 1.0 McFarland standard. The inoculum was prepared by 1:100 dilution with Middlebrook 7H9 broth supplemented with 10% oleic albumin dextrose catalase (OADC). Then, 100 µL of this inoculum was pipetted into a 96-well drug-containing plate (Baso, Zhuhai, China). The plates are inoculated at 37.0°C in an incubator with a water reservoir. The growth of bacteria was monitored at day 10 after inoculation. If there was no visible growth in the control well, the plate was incubated for an additional 4 days or 11 days. The interpretation of MIC results followed the manufacturer’s instruction. The determination as to whether an isolate was susceptible or resistant to a certain drug was based on the breakpoint concentrations for the respective drugs. The corresponding breakpoint concentrations were 1.0 mg/L for RIF, 0.125 mg/L for isoniazid (INH), 4.0 mg/L for EMB, 2.0 mg/L for streptomycin (STR), 4.0 mg/L for amikacin (AMK), 0.5 mg/L for capreomycin (CAP), and 1.0 mg/L for levofloxacin (LVX), respectively.

### TBseq test

#### Sample pretreatment

##### Sputum and pus samples

We initiated the sample pretreatment by adding an equal volume of NALC-NaOH preservation solution or dithiothreitol solution to the original sample volume, followed by a 1-minute vortex mixing to ensure homogeneity. A 10-mL aliquot of this mixture was then centrifuged at 4,000 rpm for 5 minutes. Carefully discard the supernatant without disturbing the pellet. Resuspend the pellet in 500 µL of PBS solution. Add lysozyme and mutanolysin, ensuring thorough mixing. Incubate the mixture at 30℃ for 15 minutes to facilitate cell lysis. After incubation, add proteinase K and 0.05-mm zirconia beads. Subject the mixture to mechanical disruption using a bead mill according to the manufacturer’s instructions to further disrupt the cells and release nucleic acids.

##### CSF, BALF, urine, pleural effusion, and ascites

These samples were processed by directly centrifuging a 10-mL aliquot at 4,000 rpm for 5 minutes, after which the pellet was handled according to the protocol optimized for sputum samples.

##### Tissue samples

A small piece of tissue was sampled and mixed with an equal volume of tissue preservation solution and GA buffer and then incubated at 56℃ in a water bath for 15 to 30 minutes. After incubation, lysozyme and lysostaphin were added, and the mixture was thoroughly blended before proceeding with the same downstream procedures as for liquid samples, including bead beating and enzymatic treatments.

### DNA extraction and purification

Genomic DNA was extracted from the 500-µL pretreated clinical samples, using the QIAamp DNA Microbiome Kit (QIAGEN, Canada), following the manufacturer’s guidelines. The purification process involved the use of magnetic beads, and the purified DNA was eluted in 30 µL of nuclease-free water. DNA concentration was measured using a Qubit 4 Fluorometer (Thermo Fisher Scientific, USA). The minimum DNA concentration required for TBseq test is 0.2 ng/µL. A non-template control was included as a negative control to ensure the absence of contaminating nucleic acids during extraction.

### Construction of the TBseq test panel

We developed a comprehensive panel, TBseq test, for the identification of eight *M.tb* species and 168 non-tuberculous mycobacteria (NTM) species, as well as for the detection of resistance to 16 anti-TB drugs. The panel targets 21 genes associated with drug resistance, informed by databases such as TBDReaMDB, MUBII-TB-DB, and RESEQTB.ORG (25–27), and a literature review of studies published from January 1970 to June 2020.

### Multiplex PCR amplification

A multiplex polymerase chain reaction (PCR) was performed to amplify all genetic loci, with primers designed to yield amplicons ranging from 469 to 1,028 base pairs (Table S1). The PCR reaction mixture contained KAPA HiFi HotStart Ready Mix (Roche Sequencing and Life Sciences, USA), primers, GC Enhancer (New England BioLabs, USA), and purified DNA. The PCR products were purified using magnetic beads for subsequent barcode PCR.

### Library preparation and sequencing

The barcode PCR-generated DNA products were processed for library preparation using the Ligation Sequencing Kit 1D (SQK-LSK110). The library was prepared following the manufacturer’s protocol, including end-repair, ligation of sequencing adaptors, and purification. The library was quantified using a Qubit 4 Fluorometer, and a 100-ng aliquot was loaded into a R9.4.1 flow cell for the MinION platform.

### Sequencing data analysis workflow

Raw sequencing data were processed and converted into FASTQ format using Guppy (version 5.0.12). High-quality reads were trimmed of adapters and barcodes and aligned against the NanoTargetDB using Minimap2 (version 2.24) for microbial species identification. Drug resistance profiling was performed by comparing query sequences against the *M.tb* reference genome (H37Rv, accession number NC_000962.3). Variants were detected with VarScan2 (version 2.24) and annotated with SnpEff (version 5.1e). The variant profiles were cross-referenced with the TBDB repository, TBDReaMDB, MUBII-TB-DB, RESEQTB.ORG, and the WHO-endorsed CRyPTIC catalogue for resistance information.

### Report generation and data presentation

The TBseq test Web Application compiles a comprehensive report detailing the sample analysis, methodology, quality estimates, and results of species identification and drug resistance profiling. Reports are generated in docx or pdf formats and can be directly downloaded from the application interface, ensuring efficient dissemination of results.

### Real-time fluorescence quantitative PCR

For the identification of *M.tb*, we utilized real-time fluorescence quantitative PCR (qPCR). The clinical samples underwent DNA extraction using the same methodology as for nanopore sequencing. The ABI 7500 fluorescence qPCR instrument (Thermo Fisher Scientific, USA) was employed to test the extracted DNA samples for the MTB-specific multicopy repeat insertion sequence *IS6110* and the *Mycobacterium* spp. shared gene *hsp65*. A positive qPCR result was defined as the detection of amplification curves for both the hsp65 and IS6110 targets. The hsp65 gene is ubiquitously expressed in mycobacteria and serves as a general marker for the *Mycobacterium* genus, while the IS6110 insertion sequence is specific to the MTBC. A positive result of both targets indicates the presence of MTBC, which is considered diagnostic of MTB infection. In contrast, a positive result for hsp65 alone, with a negative IS6110 result, suggests the presence of a NTM, as NTMs typically do not contain the IS6110 element but may express hsp65 (28–31).

### Turnaround time assessment

The turnaround time for generating a TBseq test report from a clinical sample using the MinION platform was assessed by tracking the time from sample receipt to the final report generation. This included all stages of the process, from DNA extraction and target enrichment to library preparation, quality control, sequencing, and data analysis. We recorded the time taken for each step of the workflow. The assessment was conducted under normal operating conditions to reflect the routine performance of the TBseq test in a clinical setting. After subtracting negative and positive controls, a maximum of 45 clinical samples can be tested per batch of the TBseq assay protocol and turnaround time is calculated based on processing the entire batch.

### Cost assessment

All cost analyses were based on standardized protocols and actual costs incurred during the study, which in this case refers to direct costs: only the costs of reagents and consumables were included. Prices for these items were obtained from manufacturers or local suppliers and recorded at the time of purchase. The total cost per sample was derived by adding the average costs of library preparation, quality control, and sequencing. The average cost per sample was calculated based on a lot size of 45 samples per run for the MinION workflow.

### Statistical analysis

The statistical analysis for this study was conducted using R statistical software (version 4.2.3). The performance estimates of the diagnostic tests, including sensitivity, specificity, PPV, NPV, and AUC were calculated using the R package reportROC. Sensitivity estimates were compared among those clinically diagnosed (“diseased”), while specificity was compared among those not diagnosed with TB (“non-diseased”). Paired data comparisons were performed using McNemar’s test via the R package rstatix, while NPV and PPV were compared using chi-squared tests or Fisher’s exact tests using R package rcompanion. Given the multiple comparisons that the authors provided, Bonferroni correction was used. AUC comparisons were compared employing DeLong’s test, available in the R package pROC. Statistical significance was defined by *P* values of less than 0.05.

## RESULTS

### Patient characteristics and study overview

**Fig 1:**
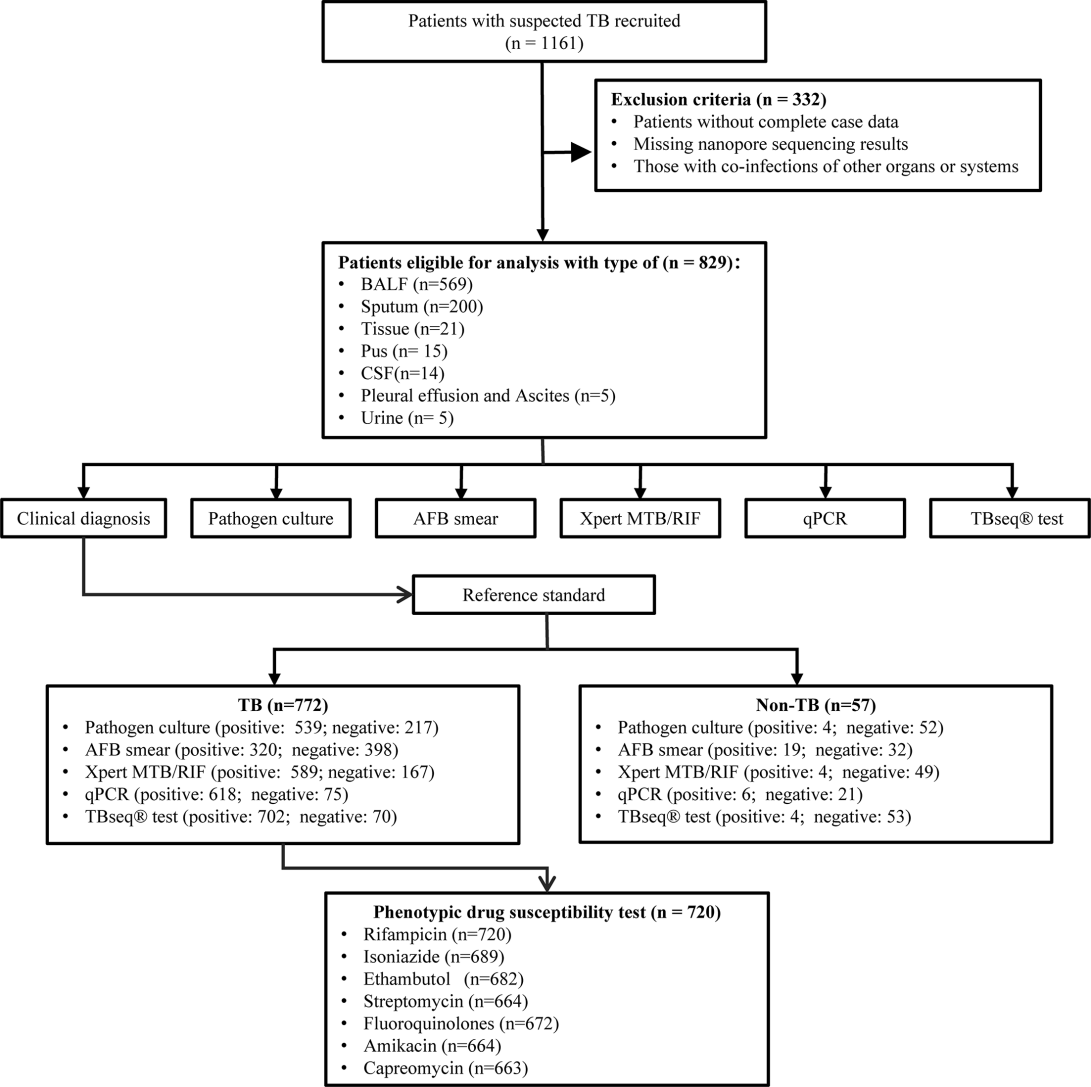
Flowchart showing the classification of patients included in the present study. The flow chart shows the design of the entire study and the number of samples used. AFB, acid-fast bacillus; TB, tuberculosis; BALF, bronchoalveolar lavage fluid; CSF, cerebrospinal fluid.

In this study, 1,161 patients with suspected TB infection were recruited from six designated TB hospitals. After exclusion criteria were applied, 829 patients were included for subsequent analysis. Clinical samples were collected from these patients and subjected to various diagnostic methods, including culture, AFB smear, Xpert MTB/RIF Ultra, and qPCR, for TB identification. The clinical diagnosis was used as the gold standard to compare the sensitivity and specificity of each diagnostic method. For patients clinically diagnosed with TB, further pDST was performed to evaluate the performance of the TBseq test in predicting drug resistance. The study flow is depicted in Fig. 1. The median age of the TB patients was 47 years, with a pronounced male predominance, representing 64.25% (*n* = 496) of the total TB patients: 17.6% (*n* = 136) had a low body mass index (BMI) (<18 kg/m²), 14.12% (*n* = 109) had diabetes mellitus, and 4.15% (*n* = 32) were diagnosed with HIV infection; 22.67% had a previous history of TB, while 3.11% reported TB exposure. Sample collection primarily consisted of BALF, making up 67.88% (*n* = 524) of the samples, with sputum samples also commonly collected at 24.87% (*n* = 192). Detailed demographic and sample type distributions are presented in Table 1.

**TABLE 1:** Clinical characteristics of the included patients

	Overall	TB	Non-TB
Total	829	772	57
Age, median (range), yr	48 (8–100)	47 (8–100)	61 (16–82)
Gender, *n* (%)			
Men	512 (61.76)	496 (64.25)	16 (28.07)
Women	317 (38.24)	276 (35.75)	41 (71.93)
BMI, median (range)	20.1 (12.57, 31.63)	20.28 (12.57, 31.63)	18.75 (14.02–26.4)
Underweight (BMI < 18.5), *n* (%)	158 (19.06)	136 (17.62)	22 (38.60)
Normal body weight (18.5 ≤ BMI ≤ 23.9), *n* (%)	319 (38.48)	294 (38.08)	25 (43.86)
Overweight (BMI > 23.9), *n* (%)	75 (9.05)	72 (9.33)	3 (5.26)
Unknown, *n* (%)	277 (33.41)	270 (34.97)	1 (1.75)
Combined diabetes, *n* (%)			
Yes	110 (13.27)	109 (14.12)	54 (94.74)
No	628 (75.75)	574 (74.35)	1 (1.75)
Unknown	91 (1.09)	89 (11.53)	2 (3.51)
Co-HIV infection, *n* (%)			
Yes	32 (3.86)	32 (4.15)	54 (94.74)
No	704 (84.92)	650 (84.20)	0
Unknown	91 (10.98)	89 (11.53)	3 (5.26)
Place of residence, *n* (%)			
City	282 (34.02)	258 (33.42)	24 (42.11)
Countryside	456 (55.01)	425 (55.05)	31 (54.39)
Unknown	91 (10.98)	89 (11.53)	2 (3.51)
History of previous TB, *n* (%)			
Yes	178 (21.47)	175 (22.67)	3 (5.26)
No	526 (63.45)	503 (65.16)	23 (40.35)
Unknown	125 (15.08)	94 (12.18)	31 (54.39)
History of TB exposure, *n* (%)			
Yes	25 (3.02)	24 (3.11)	1 (1.75)
No	712 (85.89)	659 (85.36)	53 (92.98)
Unknown	92 (11.10)	89 (11.53)	3 (5.26)
Ethnic group, *n* (%)			
Han	639 (77.08)	591 (76.55)	48 (84.21)
Other	99 (11.94)	92 (11.92)	7 (12.28)
Unknown	91 (10.98)	89 (11.53)	2 (3.51)
Sample type, *n* (%)			
BALF	569 (68.64)	524 (67.88)	45 (78.95)
Sputum	200 (24.13)	192 (24.87)	8 (14.04)
Tissue	21 (2.53)	19 (2.46)	2 (3.51)
Pus	15 (1.81)	14 (1.81)	1 (1.75)
CFS	14 (1.69)	14 (1.81)	0
Urine	5 (0.60)	5 (0.65)	0
Pleural effusion and ascites	5 (0.60)	4 (0.52)	1 (1.75)

### Diagnostic performance of *M.tb* detection assays

**Fig 2:**
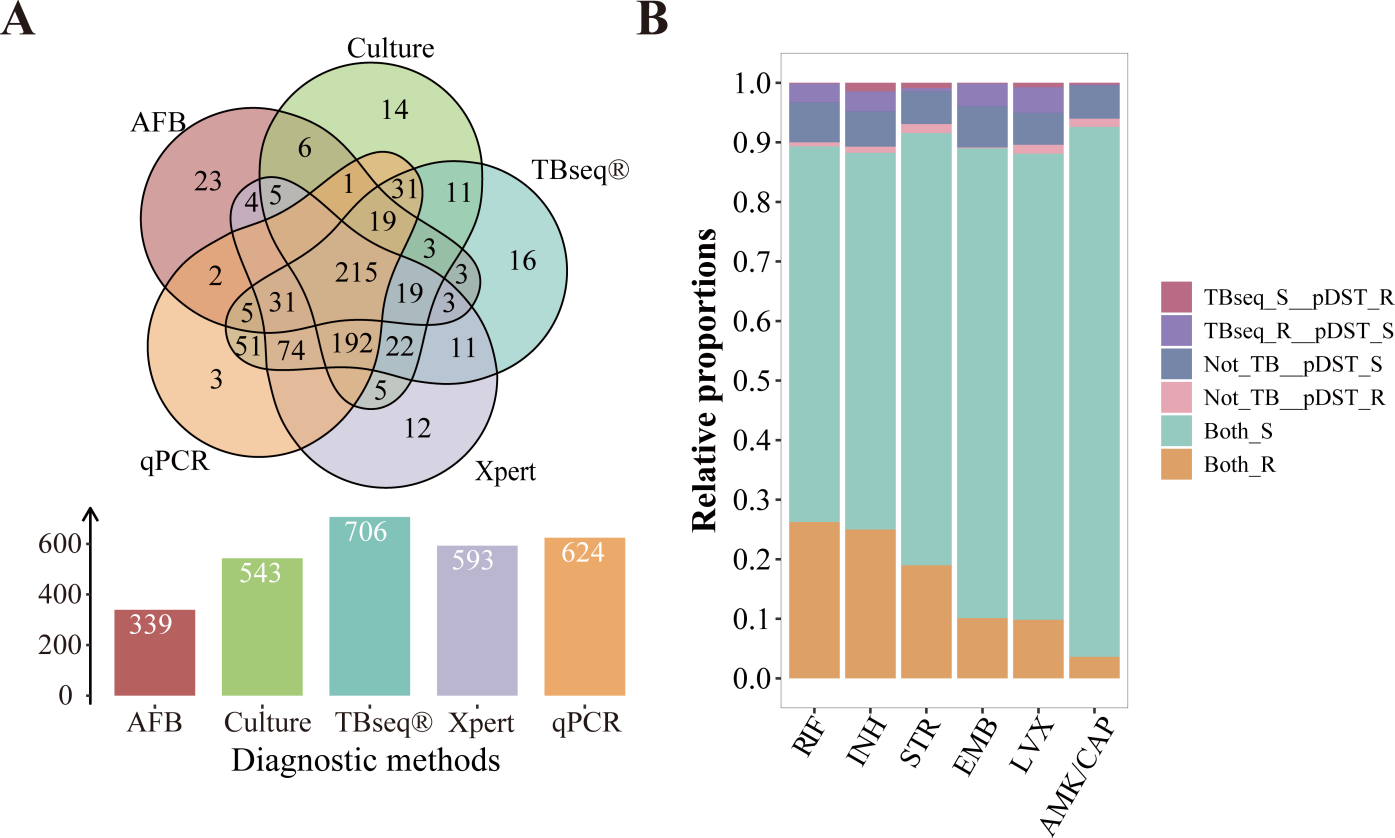
Comparison of detected TB-positive cases by various methods and correlation between TBseq test resistance profiling and pDST results. (**A**) Number of positive cases identified by different methods. The *x*-axis represents various detection methods, whereas the *y*-axis denotes the count of TB patients identified through each method. (**B**) Comparative analysis of TBseq test-derived resistance determination vs pDST results. The *x*-axis indicates different anti-tubercular drugs, and the *y*-axis shows the proportion of different resistance outcomes. Abbreviations: RIF (rifampicin), INH (isoniazid), STR (streptomycin), EMB (ethambutol), LVX (levofloxacin), AMK (amikacin), CAP (capreomycin). Both_R: concordant resistant results by both TBseq test and pDST; Both_S: concordant susceptible results by both TBseq test and pDST; TBseq test_S_pDST_R: TBseq test indicates susceptibility, whereas pDST shows resistance; TBseq test_R_pDST_S: TBseq test indicates resistance, whereas pDST shows susceptibility; Not_TB_pDST_R: TBseq test does not diagnose TB, yet pDST indicates resistance; Not_TB_pDST_S: TBseq test does not diagnose TB, yet pDST indicates susceptibility.

We assessed the diagnostic accuracy of five assays, including *M.tb* culture, TBseq test, qPCR, Xpert MTB/RIF Ultra, and AFB smear, for identifying *M.tb* in 829 eligible subjects. The reference standard for the diagnosis of TB was clinical diagnosis. Clinical diagnosis confirmed TB in 772 patients, while the remaining 57 were non-TB cases. The results are summarized in Fig. 2A; Table S2. Specifically, a total of 812 *M*.*tb* cultures were completed, yielding 543 positive and 269 negative results. TBseq test results were available for all 829 subjects, with 706 positive and 123 negative findings. qPCR results were obtained for 720 subjects, with 624 positive and 96 negative outcomes. Xpert MTB/RIF Ultra was performed on 809 subjects, resulting in 593 positive and 216 negative results. AFB smears were evaluated for 769 subjects, identifying 339 positive and 430 negative cases. Only TBseq test yielded complete test results for all 829 patients. The other four methods suffered from incomplete data due to various reasons. These reasons encompassed technical issues or equipment malfunctions, insufficient sample volume, failure of sample quality control, and human operational errors. Using clinical diagnosis as the reference standard, the detection rates among the 772 patients with confirmed TB were as follows: *M.tb* culture identified 539 cases, TBseq test identified 702 cases, qPCR identified 618 cases, Xpert MTB/RIF Ultra identified 589 cases, and AFB smear identified 320 cases. Among the 57 non-TB patients, the following number of negative results were recorded for each diagnostic method: *M.tb* culture in 52 cases, TBseq test in 53 cases, qPCR in 21 cases, Xpert MTB/RIF in 4 cases, and AFB smear in 19 cases. TBseq test demonstrated the highest performance with a sensitivity of 90.9% (95% confidence interval [CI]: 88.9%–93.0%), and an AUC of 92.0% (95% CI: 0.876–0.963), outperforming conventional culture, Xpert MTB/RIF Ultra, qPCR, and AFB smear, which had varying sensitivities and specificities as detailed in Table 2.

**TABLE 2:** Diagnostic accuracy of the five tests for the diagnosis of TB

Test	TP	FP	TN	FN	Sensitivity % (95% CI^*a*^)	Specificity % (95% CI)	PPV^*b*^ % (95% CI)	NPV^*c*^ % (95% CI)	AUC^*d*^ (95% CI)
TBseq Test	702	4	53	70	90.9 (88.9–93.0)	93.0 (86.4–99.6)	99.4 (98.9–100.0)	43.1 (34.3–51.8)	0.92 (0.876–0.963)
Xpert MTB/RIF	589	4	49	167	77.9 (75.0–80.9)	92.5 (85.3–99.6)	99.3 (98.7–100.0)	22.7 (17.1–28.3)	0.852 (0.801–0.902)
qPCR	618	6	21	75	89.2 (86.9–91.5)	77.8 (62.1–93.5)	99.0 (98.3–99.8)	21.9 (13.6–30.1)	0.835 (0.745–0.925)
Culture	539	4	52	217	71.3 (68.1–74.5)	92.9 (86.1–99.6)	99.3 (98.5–100.0)	19.3 (14.6–24.0)	0.821 (0.771–0.871)
AFB Smear	320	19	32	398	44.6 (40.9–48.2)	62.7 (49.5–76.0)	94.4 (91.9–96.8)	7.4 (5.0–9.9)	0.537 (0.452–0.621)

^*a*^ Confidence interval.

^*b*^ Positive predictive value.

^*c*^ Negative predictive value.

^*d*^ Area under the receiver operating characteristic curve.

An in-depth statistical comparison of the performance estimates revealed a notable trend in sensitivity, with TBseq test showing the highest, followed by qPCR, Xpert MTB/RIF Ultra, culture, and AFB smear with the lowest (*P* < 0.001 for all comparisons; Table S3). Specificity varied, with TBseq test maintaining the highest level, but without statistical significance compared to culture, Xpert MTB/RIF Ultra, and qPCR. TBseq test’s PPV significantly exceeded that of AFB smear (*P* < 0.001), though not significantly different from the other three methods. TBseq test also showed a significantly higher NPV than culture, Xpert MTB/RIF Ultra, AFB smear, and qPCR (*P* < 0.001, *P* < 0.001, *P* < 0.001, and *P* < 0.01, respectively). AUC comparisons mirrored NPV results, with TBseq test notably superior to other four methods (*P* < 0.001, *P* < 0.001, *P* < 0.001, and *P* < 0.05, respectively).

Regarding the culture methods, our study utilized both the MGIT960 and the LJ culture methods. Given that MGIT960 is generally more sensitive than the LJ method, we conducted a separate analysis for each. The results indicated that the MGIT960 liquid culture method had higher sensitivity, specificity, NPV, PPV, and AUC values compared to the LJ solid culture method, with a significant difference observed only in sensitivity (*P* < 0.001) (Table S4). When comparing the results of MGIT960 and LJ culture methods with other diagnostic methods, the majority of the comparative outcomes aligned with those of the combined culture results (Table S3). However, there were instances where discrepancies were noted in relation to the previously reported combined culture outcomes. For instance, the AUC value for the combined culture results was significantly lower than that of TBseq test. A statistically significant difference in AUC values was detected between the LJ culture and TBseq test (*P* < 0.001), whereas no significant differences in AUC values were observed between MGIT960 and TBseq test.

### Comparison of the accuracy of BALF samples and sputum samples for the diagnosis of TB

We collected and tested seven different types of samples, predominantly BALF (*n* = 569) and sputum (*n* = 272), which are also the most commonly used samples in clinical practice. Consequently, we separately evaluated the performance of various detection methods for these two sample types. Generally, for BALF samples, the TBseq test exhibited the highest performance across all estimates, with a sensitivity of 90.3% (95% CI: 87.7%–92.8%), specificity of 95.6% (95% CI: 89.5%–100.0%), PPV of 99.6% (95% CI: 99.0%–100.2%), NPV of 45.7% (95% CI: 35.7%–55.8%), and AUC of 0.929 (95% CI: 0.886–0.972) (Table S5). In contrast, for sputum samples, qPCR demonstrated the overall best performance, followed by the TBseq test (Table S6).

A comparative analysis of the performance of different methods on the two sample types revealed that only Xpert MTB/RIF Ultra assay had superior overall performance on BALF samples, with a significantly higher sensitivity compared to sputum samples (Table S7, *P* = 0.02962). Conversely, the other four methods exhibited higher overall performance on sputum samples compared to BALF. Notably, the culture method showed a significantly higher AUC for sputum samples (*P* = 0.006635). Similarly, the TBseq test yielded significantly higher sensitivity and AUC for sputum samples (*P* = 0.0247 and *P* = 0.00644, respectively), as did qPCR (*P* = 0.001027 and *P* = 0.0022, respectively). Additionally, the sensitivity of the AFB smear test was significantly greater for sputum samples than for BALF (*P* < 0.001).

### Comparative analysis of TBseq test and pDST

We conducted a comprehensive assessment of drug resistance in 720 TB patients using pDST for seven first-line and common second-line anti-TB drugs: RIF, INH, EMB, STR, AMK, CAP, and LVX. For drug resistance, the reference standard was pDST. Resistance to at least one drug was observed in 282 patients, while for 438 patients, no resistance was detected for any of the drugs tested (Table S8). Based on the pDST results obtained from the TB patients, 27.08% of patients were resistant to RIF, 27.43% to INH, 10.41% to EMB, 21.39% to STR, and 12.05% to LVX, and fewer than 5% of patients were resistant to AMK or CAP. According to the WHO criteria, the study identified 103 patients (14%) with MDR-TB, 28 patients (3.9%) with poly-drug resistant TB, and 46 patients (6.4%) with Pre-XDR-TB.

We further compared the efficacy of TBseq test against pDST (Table 3). Resistance to RIF was concordantly identified by Xpert MTB/RIF Ultra, TBseq test, and pDST in 151 patients. Notably, TBseq test in conjunction with pDST detected RIF resistance in an additional 38 patients where Xpert MTB/RIF Ultra was negative. Conversely, Xpert MTB/RIF Ultra along with pDST identified RIF resistance in three patients where TBseq test was negative (Fig. S1). TBseq test outperformed all methods in detecting RIF resistance, achieving a sensitivity, specificity, PPV, and NPV of 99.5%, 95.2%, 89.2%, and 99.8%, respectively, and an AUC of 97.3%. Moreover, TBseq test delivered exceptional results in the detection of resistance to INH, EMB, STR, AMK, CAP, and LVX, maintaining high sensitivity and specificity. For instance, the sensitivity and specificity for INH were 94.5% and 95.0%, respectively, with an AUC of 94.7%; for EMB, these values were as high as 98.6% and 95.4%, respectively, with an AUC of 97.0%.

**TABLE 3:** Comparison of the performance of different methods for the identification of TB drug resistance

Drugs	RIF (Xpert)		RIF		INH		EMB		STR		AMK		CAP		LVX	
pDST	R	S	R	S	R	S	R	S	R	S	R	S	R	S	R	S
R^*a*^	154	15	189	1	172	10	69	1	126	6	24	0	18	1	66	5
S^*b*^	28	356	23	454	23	436	26	538	4	482	2	591	8	590	29	526
Sensitivity % (95% CI^*c*^)	91.1 (86.8–95.4）		99.5 (98.4–100)		94.5 (91.2–97.8）		98.6 (95.8–100）		95.5 (91.9–99.0）		100.0 (82.8–100.0)		94.7 (84.7–100.0)		93.0 (87.0–98.9)	
Specificity % (95% CI)	92.7 (90.1–95.3）		95.2 (93.3–97.1)		95.0 (93.0–97.0）		95.4 (93.7–97.1）		99.2 (98.4–100.0）		99.7 (98.6–99.9)		98.7 (97.7–99.6)		94.8 (92.9–96.6)	
PPV^*d*^ % (95% CI)	84.6 (79.4–89.9)		89.2 (85.0–93.3)		88.2 (83.7–92.7）		72.6 (63.7–81.6）		96.9 (94.0–99.9）		92.3 (73.4–98.7)		69.2 (51.5–87.0)		69.5 (60.2–78.7)	
NPV^*e*^ % (95% CI)	96.0 (94.0–98.0)		99.8 (99.3–100）		97.8 (96.4–99.1）		99.8 (99.5–100.2）		98.8 (97.8–99.7）		100.0 (99.1–100.0)		99.8 (99.5–100.2)		99.1 (98.2–99.9)	
AUC^*f*^ (95% CI)	0.919 (0.885–0.954）		0.973 (0.959–0.988)		0.947 (0.921–0.974）		0.970 (0.947–0.992）		0.973 (0.951–0.995）		0.998 (0.996–1.000)		0.967 (0.912–1.000)		0.939 (0.900–0.978)	

^*a*^ Sensitive to drug.

^*b*^ Resistant to drug.

^*c*^ Confidence interval.

^*d*^ Positive predictive value.

^*e*^ Negative predictive value.

^*f*^ Area under the receiver operating characteristic curve.

### Consistency and discordance between TBseq test and pDST resistance test results

**Fig 3:**
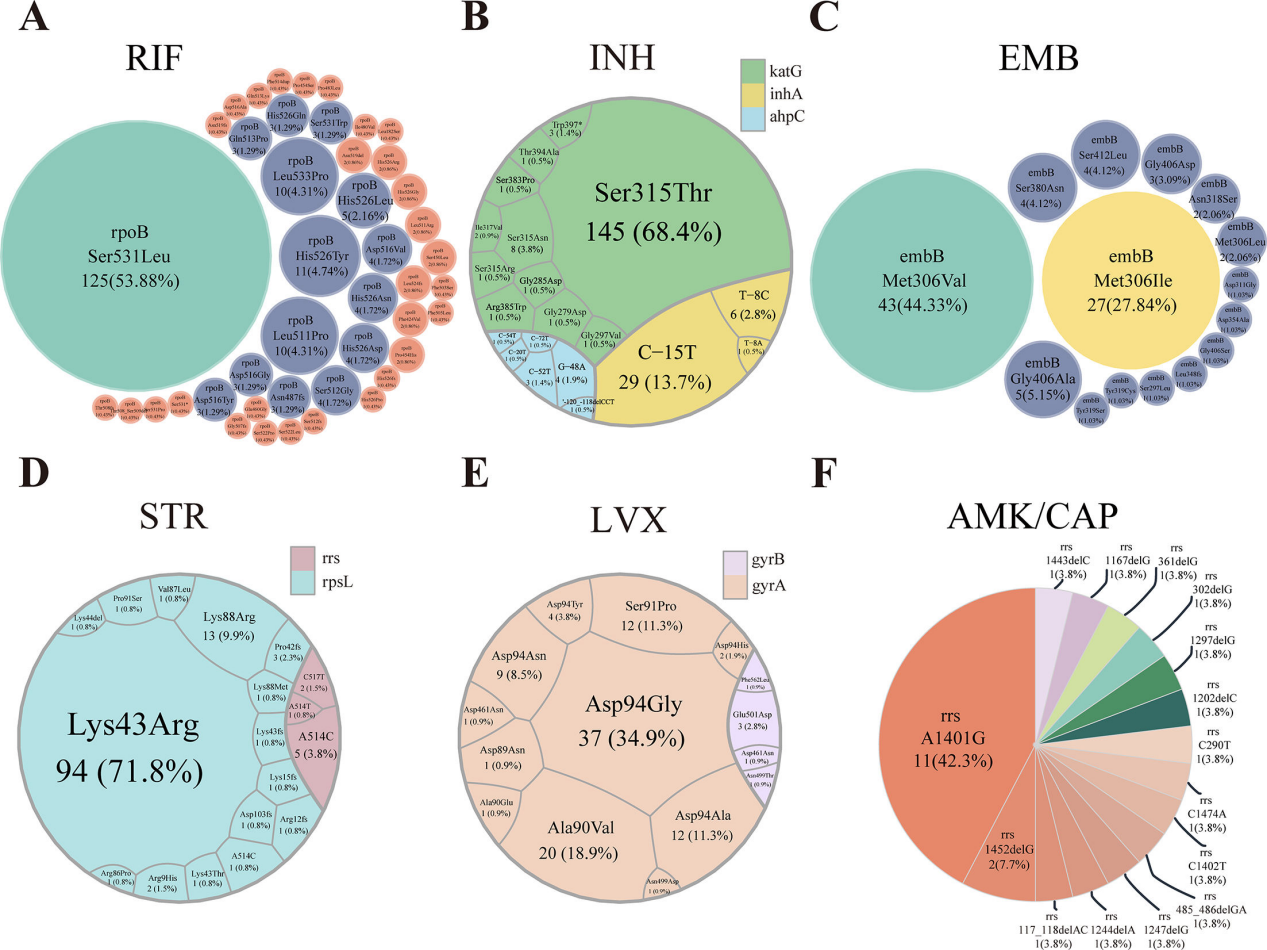
Visualization of drug resistance mutations. (**A**) Bubble chart depicting mutations associated with RIF resistance (*rpoB*). Bubble sizes reflect the frequency of mutations within the *rpoB* gene linked to RIF resistance. (**B**) Voronoi diagram illustrating mutations linked to INH resistance (*katG*, *inhA*, a*aphC*). The size of each section in the diagram corresponds to the relative proportion of mutations in these genes among all identified mutations contributing to INH resistance. (**C**) Bubble chart representing mutations related to EMB resistance (*embB*). (**D**) Voronoi diagram highlighting mutations associated with STR resistance (*rrs*, *rpsL*). (**E**) Voronoi diagram showing mutations connected to LVX resistance (*gyrA*, *gyrB*). (**F**) Pie chart exhibiting mutations relevant to AMK/CAP resistance (*rrs*). Color key for bubble charts: red represents proportions from 0% to 1%; dark blue signifies proportions from 1% to 20%; yellow indicates proportions from 20% to 40%; green denotes proportions from 40% to 100%.

A high level of concordance was found between TBseq test and pDST results, with nearly 90% of patients showing consistent results for each drug (Fig. 2B). In-depth analysis of the drug-resistant genetic profiles of *M.tb* was conducted, and the main findings are summarized in Table S9; Fig. 3. Specially, 45 distinct *rpoB* mutation variants were identified, with *rpoB* Ser531Leu predominating in 125 cases (53.88%). Double mutations within the rpoB gene were noted in 16 cases, and one case had triple mutations (Table S10). INH resistance was predicted in 194 cases using TBseq test, primarily due to mutations in *katG*, *inhA*, and *ahpC. katG* Ser315Thr was the most frequent mutation, found in 145 cases (68.4%) (Table S9). EMB resistance was detected in 95 cases, attributed to 17 distinct *embB* mutations. The most common was *embB* Met306Val (43 cases, 44.33%), followed by *embB* Met306Ile (27 cases, 27.84%). STR resistance was identified in 130 cases, primarily due to *rpsL* mutations, with *rpsL* Lys43Arg being the most common (94 cases, 71.8%). LVX resistance was found in 94 cases, mainly associated with *gyrA* mutations (88 cases, 94.5%). The most common *gyrA* mutations were Asp94Gly (37 cases, 34.9%). Coexistence of multiple mutations in *gyrA* and *gyrB* was observed in several cases. All 26 patients with AMK/CAP resistance had *rrs* mutations, with A1401G being the most common (11 cases, 42.3%). Although pDST was not performed for pyrazinamide, TBseq test predicted resistance in 71 cases, associated with 59 diverse *pncA* mutations (Fig. S2).

Discrepancies between TBseq test and pDST were noted for RIF (24 cases), INH (33 cases), EMB (38 cases), STR (16 cases), AMK/CAP (11 cases), and LVX (49 cases) (Table S11). A prevalent pattern was pDST indicating susceptibility while TBseq test indicated resistance, particularly for RIF (23 cases), INH (23 cases), EMB (37 cases), and LVX (41 cases).

### Turnaround time and cost assessment

Given the strong performance of TBseq test in identifying *M.tb* and predicting drug resistance, we estimated the cost of diagnosis and turnaround time, which are important components in assessing its potential economic impact on diagnostic services. The mean duration from DNA extraction to final report generation using TBseq test is approximately 16 hours (Table S12), suggesting that results can be delivered within a 2-day timeframe in laboratories operating on an 8-hour daily schedule. Economically, the estimated per-sample testing cost for TBseq test is approximately US$55.7 (Table S12). This rapid turnaround and defined cost structure highlight TBseq test’s clinical feasibility and potential efficiency, facilitating timely therapeutic interventions and public health responses.

## DISCUSSION

In this retrospective study, we enrolled 829 Chinese patients with suspected TB for a comprehensive analysis of the diagnostic performance of five methods, including *M.tb* culture, Xpert MTB/RIF Ultra, TBseq test, qPCR, and AFB smear microscopy. Our results indicated that TBseq test displays the highest diagnostic accuracy. These findings underscore the potential of optimized nanopore sequencing as a promising diagnostic tool for TB detection. Moreover, we conducted a comparative analysis of the diagnostic results of pDST and TBseq test for seven anti-TB drugs. Our data reveal that TBseq test’s sensitivity and specificity in diagnosing resistance to RIF, INH, EMB, STR, LVX, AMK, and CAP all exceeded 90%. Furthermore, our data imply a consistency of resistance test results between TBseq test and pDST, which are consistent with previously reported resistance profiles (32, 33). Thus, TBseq test is a promising predictive tool for drug resistance that meets the need for rapid and precise resistance profiling.

Our study capitalized on the diversity of sample types to assess diagnostic performance variability. Sputum and BALF, as the most frequently used samples, consistently showed the high diagnostic performance with TBseq test. Comparative analysis revealed that, except for *M.tb* culture, the sensitivity of the other four methods for sputum samples was significantly higher than that for BALF samples (*P* = 0.0247, *P* = 0.02962, *P* = 0.001027, and *P* < 0.001, respectively) (Table S7). This suggests that sputum may offer superior diagnostic sensitivity in the context of molecular diagnostic techniques like the TBseq test. In terms of AUC, culture, TBseq test, and qPCR exhibited better diagnostic capabilities for sputum than for BALF samples (*P* = 0.006635, *P* = 0.00644, *P* = 0.0022, and *P* < 0.001, respectively). For less commonly used samples such as pus, CSF, tissue, urine, pleural effusion, and ascites, TBseq test showed remarkable sensitivity (Table S13). Notably, TBseq test accurately identified 13 out of 14 (92.9%) pus samples, all four cases with pleural effusion and ascites, and all five cases with urine samples. Tissue samples also showed a high sensitivity of (16/19, 84.2%) for TBseq test. In the case of CSF, which typically presents lower bacterial loads, TBseq test detected seven out of 14 cases, maintaining the highest detection rate among the techniques compared, in line with Zhou et al. (34). These collective findings underscore the robust diagnostic performance of the TBseq test across a variety of sample types, including those with lower bacterial loads. This further underscores the potential of the TBseq test as a versatile tool for TB detection in clinical practice. The high sensitivity and specificity of the TBseq test in sputum samples, in particular, position it as a preferred diagnostic method, especially in settings where resources are constrained.

We included patients with symptoms suggestive of active TB at six specialized hospitals across China from September 2022 to July 2023. Participants were primarily residents of the cities or regions where the hospitals are located (Table S2). For example, patients visiting the Hebei Chest Hospital were predominantly from Hebei province and its surrounding areas. A small number of patients (approximately 2%) were referred from other regions but were still included in the study due to their ongoing treatment at these specialized centers. However, it is important to note that the study population in this research has a higher prevalence of TB compared to the general population of presumed TB patients. This higher prevalence is attributed to the specific context of our study. Patients were recruited from six designated TB hospitals, where individuals with more severe TB symptoms or those referred from other hospitals are more likely to present. Additionally, these specialized hospitals have advanced TB diagnostic technologies and experienced medical staff, which may contribute to the higher TB prevalence observed in our study population. However, the findings from this group provide valuable insights into the diagnostic challenges and potential alternate sampling methods for patients with suspected TB where conventional sputum sampling is not possible.

This high prevalence can affect the interpretation of the PPV and NPV of the diagnostic tests used in our study. In a population with a very high prevalence of disease, even a poorly specific test can have a high PPV, and the NPV can be low even with a highly sensitive test (35, 36). Consequently, these estimates should be interpreted cautiously, as the observed high PPV and low NPV may not solely reflect test specificity and sensitivity. Future research should include a broader, more representative patient cohort to accurately assess test performance across varying prevalence rates. The analysis of the test performance with respect to predicting pDST results is more reasonable, and the group of patients tested is indeed more appropriate for assessing this aspect of the assay’s performance. The inclusion of a high proportion of TB cases with varying degrees of drug resistance allows for a robust evaluation of the test’s ability to predict pDST results accurately. This targeted approach ensures that the estimates, such as sensitivity and specificity, are meaningful for clinical decision-making in the context of managing drug-resistant TB.

Drug-resistant tests based on 720 TB patients demonstrated a high concordance (nearly 90%) between TBseq test and pDST results, underscoring TBseq test’s remarkable accuracy in identifying drug resistance. Collectively, TBseq test consistently showed high sensitivity (≥90%) and specificity across the assessment of resistance to these seven drugs, substantiating its robust potential and precision as a diagnostic tool for drug resistance (Table 3). TBseq test’s superior speed offers an advantage over pDST, providing rapid clinical insights that can expedite treatment and recovery. However, a minority of samples exhibited discrepancies between two methods, with 24 cases of RIF, 33 of INH, 38 of EMB, 16 of STR, two of AMK, nine of CAP, and 46 of LVX showing inconsistent results. These were categorized into TBseq test-negative but pDST-positive and TBseq test-positive but pDST-negative, presenting clinical challenges.

The relatively low incidence of TBseq test-negative but pDST-positive results may be attributed to subthreshold bacterial loads in respiratory samples, as suggested by Xu et al.’s (37) study using nanopore metagenomics. This could also result from the lack of coverage of emerging resistance mutations in our sequencing panel. Sheka et al. emphasize the importance of updating panels based on the latest literature to enhance detection rates and prevent oversights (38). Consequently, we will update our panel to align with current knowledge, aiming to improve the molecular detection of drug resistance. For cases where TBseq tests negative but pDST is positive, secondary sampling and panel updates should be considered to enhance detection accuracy.

The higher proportion of TBseq test-positive but pDST-negative results may indicate the presence of low-level drug resistance (39), where *M.tb* exhibits resistance at low drug concentrations but appears sensitive at higher concentrations (40). This poses a clinical challenge, since untreated low-level resistance could progress to full resistance or multidrug resistance, jeopardizing patient outcomes. Conventional pDST may not detect low-level resistance, necessitating more sophisticated testing methods (41). Additionally, the discrepancy could be attributed to the inherent error rate of nanopore sequencing technology (42). Furthermore, while we did not sequence the isolates used for pDST, we considered that these discrepancies could arise from differences in sample materials. Specifically, cases involving mixed, heteroresistant populations or mutations that do not contribute to resistance can lead to concordant but seemingly discrepant results. The presence of subpopulations with varying levels of drug resistance and non-contributing mutations complicates the correlation between genotypic and phenotypic data. For example, an isolate might contain both susceptible and resistant subpopulations, leading pDST to indicate susceptibility while TBseq identifies resistance-associated mutations in the minor subpopulation. Given these complexities, it is essential to integrate both genotypic and phenotypic data and validate findings through additional methods. Future studies will explore sequencing of pDST isolates to better reconcile these discrepancies and improve the reliability of drug resistance testing, ultimately enhancing clinical decision-making and patient outcomes. Clinicians should prioritize pDST results and integrate them with other clinical data for a comprehensive drug resistance assessment. For TBseq test-positive and pDST-negative cases, the TBseq test results should be taken into consideration, particularly in identifying low-level resistance, to inform appropriate treatment plans.

Collectively, for cases where TBseq test is negative but pDST is positive, secondary sampling and panel updates should be considered to enhance detection accuracy. Conversely, for TBseq test-positive and pDST-negative cases, clinicians should carefully consider the TBseq results, especially when identifying low-level resistance, to guide appropriate treatment strategies. By addressing these challenges, we aim to improve the accuracy and reliability of drug resistance testing methods, ultimately leading to better patient outcomes.

The current clinical diagnostics for TB mainly include *M.tb* culture, AFB smear, Xpert MTB/RIF Ultra, qPCR, and TBseq test, with estimated costs ranging from USD 2–70 per test. These costs can vary due to geographic location, medical institutions, and service providers, reflecting the diverse economic landscapes and healthcare infrastructures (43, 18). The time required for these tests also varies widely, from several weeks for culture to as quick as 16 hours for TBseq test. While TBseq offers rapid and sensitive results, it has several limitations. Its complexity and staff time requirements can be challenging in resource-limited settings. Additionally, it needs a stable electrical supply and powerful computers for data analysis, along with specialized training for result interpretation. Nanopore sequencing technologies often require a minimum batch size for optimal efficiency, leading to potential delays in lower-volume labs. If fewer than the maximum number of samples are processed per batch, the cost per sample increases, affecting overall cost-effectiveness.

Our multicenter study, while robust, has several limitations that should be mentioned. An important limitation of our study is the relatively low number of “non-diseased” participants, which resulted in wider CIs for specificity estimates and reduced our ability to detect differences in specificity between the tests. This limitation underscores the importance of including a larger and more balanced sample of “non-diseased” individuals in future studies to improve the precision of specificity estimates and to enhance the generalizability of our findings. Second, the samples in our study were collected from designated TB hospitals in six different provinces or regions in China. While these locations provide a diverse representation, they may not fully capture the variability in TB patient characteristics across the entire country. Therefore, future research should strive to broaden the geographical inclusion to ensure a more comprehensive and representative sample. This will help in validating the findings and ensuring their applicability to a wider population. Lastly, certain mutations associated with low-grade drug resistance may not be accurately diagnosed using the critical concentration methods for pDST. This might affect the inconsistencies observed between phenotypic and genotypic resistance in our study. To mitigate this issue, future research should focus on refining pDST methodologies to enhance their sensitivity and accuracy in detecting low-grade drug resistance mutations.

In conclusion, our retrospective study, encompassing 829 Chinese patients with suspected TB, comprehensively evaluated the diagnostic accuracy of five methodologies: *M.tb* culture, Xpert MTB/RIF Ultra, TBseq test, qPCR, and AFB smear microscopy. The TBseq test emerged as the most accurate diagnostic tool, highlighting the potential of nanopore sequencing in TB detection. Furthermore, TBseq test exhibits remarkable predictive accuracy of resistance to seven anti-TB drugs when compared to the reference standard of pDST. Collectively, this study strongly supports the integration of TBseq test into clinical practice as an efficient and accurate method for TB identification and drug resistance profiling. Further application of TBseq test-based method may lead to substantial improvements in the accuracy and the timing of TB diagnosis, hence strengthening the management of TB.

## Data Availability

Data supporting the findings of this study have been deposited in the Genome Sequence Archive (GSA) at https://ngdc.cncb.ac.cn/gsa/ under the accession number CRA022529. All other data supporting the findings of the study are available within the paper and its supplemental material.

## SUPPLEMENTAL MATERIAL

Supplemental materials jcm.01813-24-s0001.pdf Figures S1 and S2; Legends for Tables S1 to S13.

Table S1 jcm.01813-24-s0002.xlsx The targeted genes and specific sequences of primers of TBseq test.

Table S2 jcm.01813-24-s0003.xlsx The diagnostic results of each diagnostic method for the enrolled subjects.

Table S3 jcm.01813-24-s0004.xlsx Comparison of sensitivity, specificity, PPV, NPV, and AUC among different diagnostic tests.

Table S4 jcm.01813-24-s0005.xlsx The diagnostic accuracy of the MGIT 960 and LJ culture for the diagnosis of tuberculosis.

Additional supplemental tables jcm.01813-24-s0006.docx Tables S5 to S13.

ASM does not own the copyrights to Supplemental Material that may be linked to, or accessed through, an article. The authors have granted ASM a non-exclusive, world-wide license to publish the Supplemental Material files. Please contact the corresponding author directly for reuse.
