# Supplementary material for: Nanopore sequencing for precise detection of Mycobacterium tuberculosis and drug resistance: a retrospective multicenter study in China
Source: J Clin Microbiol. 2025 Mar 19;63(4):e01813-24. doi: 10.1128/jcm.01813-24 (PMC11980377; doi:10.1128/jcm.01813-24)
Supplement: Supplemental materials — Figures S1 and S2; Legends for Tables S1 to S13. [file jcm.01813-24-s0001.pdf]

# Supplementary Materials for

**Nanopore Sequencing for Precise Detection of *Mycobacterium tuberculosis* and Drug Resistance: A Retrospective Multicenter Study in China**

Shanshan Yu et al.

Corresponding author: Yu Pang, pangyupound@163.com; Liang Li, liliang69@tb123.org; Yunfei Wang, eric_wang@shengtinggroup.com

**This PDF file includes:**

Figures S1 and S2

Legends for Tables S1 to S13

**Other Supplementary Materials for this manuscript include the following:**

Tables S1 to S13

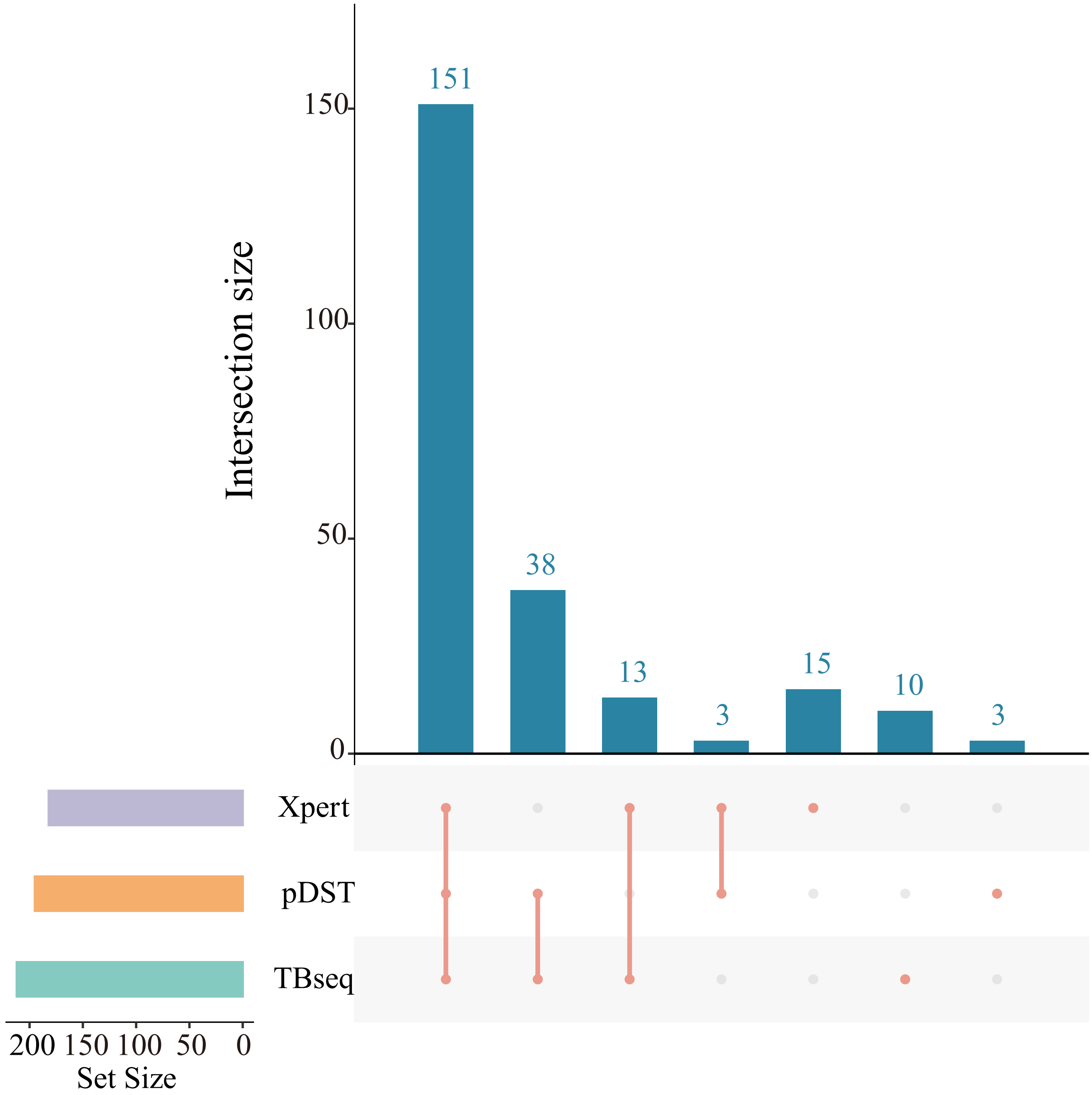
151
38
13
3
15
10
3
150
100
50
0
Intersection size
Xpert
pDST
TBseq
200 150 100 50 0
Set Size

**Figure S1** UpSet plot showing the shared rifampicin-resistance specimens among TBseq® test, pDST, and Xpert MTB/RIF.

# PZA

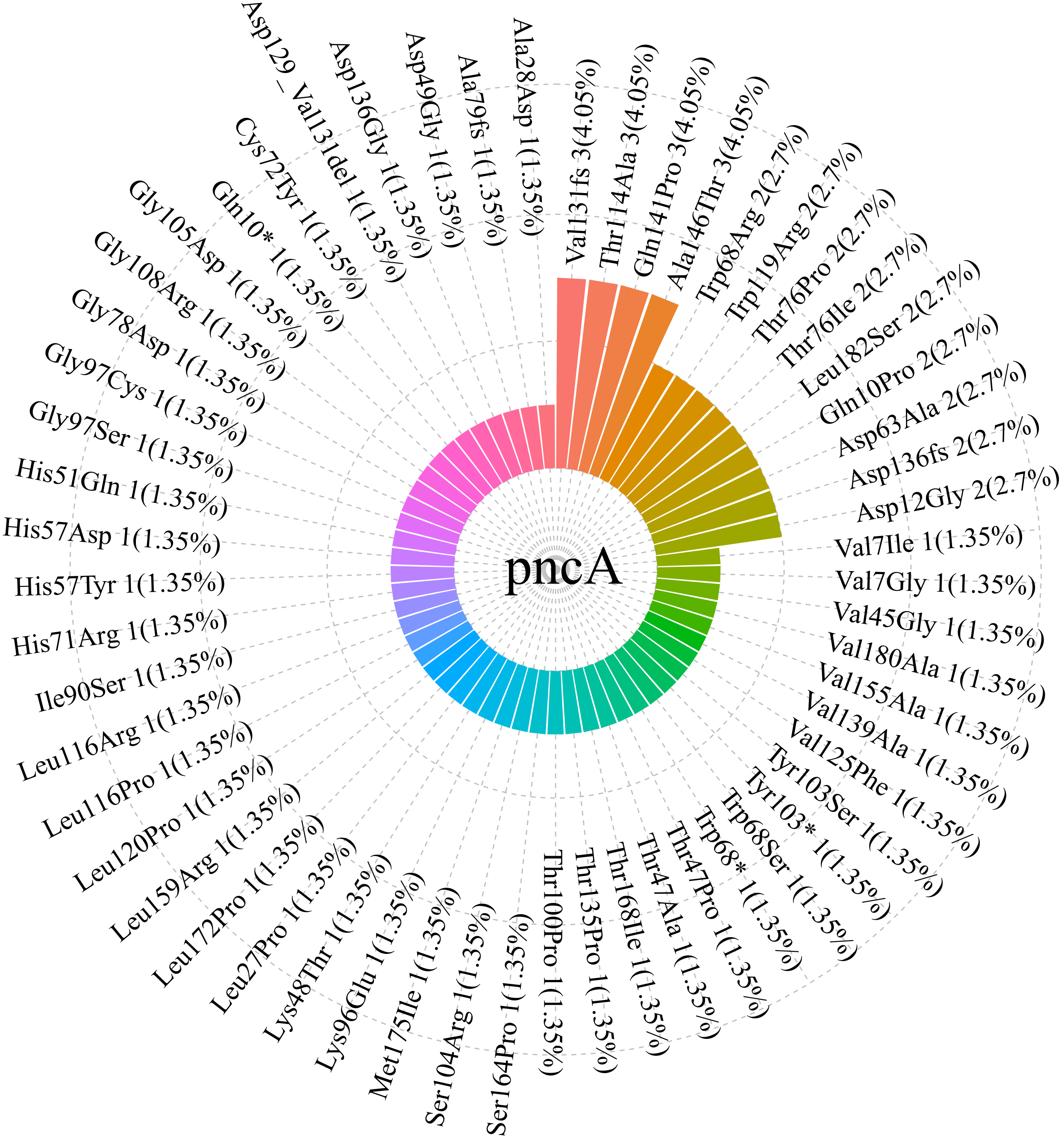
pncA
Val131fs 3(4.05%)
Thr114Ala 3(4.05%)
Gln141Pro 3(4.05%)
Ala146Thr 3(4.05%)
Trp68Arg 2(2.7%)
Trp119Arg 2(2.7%)
Thr76Pro 2(2.7%)
Thr76Ile 2(2.7%)
Leu182Ser 2(2.7%)
Gln10Pro 2(2.7%)
Asp63Ala 2(2.7%)
Asp136fs 2(2.7%)
Asp12Gly 2(2.7%)
Val7Ile 1(1.35%)
Val7Gly 1(1.35%)
Val45Gly 1(1.35%)
Val180Ala 1(1.35%)
Val155Ala 1(1.35%)
Val139Ala 1(1.35%)
Val125Phe 1(1.35%)
Tyr103Ser 1(1.35%)
Tyr103* 1(1.35%)
Trp68Ser 1(1.35%)
Trp68* 1(1.35%)
Thr47Pro 1(1.35%)
Thr47Ala 1(1.35%)
Thr168Ile 1(1.35%)
Thr135Pro 1(1.35%)
Thr100Pro 1(1.35%)
Ser164Pro 1(1.35%)
Ser104Arg 1(1.35%)
Met175Ile 1(1.35%)
Lys96Glu 1(1.35%)
Lys48Thr 1(1.35%)
Leu27Pro 1(1.35%)
Leu172Pro 1(1.35%)
Leu159Arg 1(1.35%)
Leu120Pro 1(1.35%)
Leu116Pro 1(1.35%)
Leu116Arg 1(1.35%)
Ile90Ser 1(1.35%)
His71Arg 1(1.35%)
His57Tyr 1(1.35%)
His57Asp 1(1.35%)
His51Gln 1(1.35%)
Gly97Ser 1(1.35%)
Gly97Cys 1(1.35%)
Gly78Asp 1(1.35%)
Gly108Arg 1(1.35%)
Gly105Asp 1(1.35%)
Gln10* 1(1.35%)
Cys72Tyr 1(1.35%)
Asp129_Val131del 1(1.35%)
Asp136Gly 1(1.35%)
Asp49Gly 1(1.35%)
Ala79fs 1(1.35%)
Ala28Asp 1(1.35%)

**Figure S2**: Mutational landscape of pyrazinamide resistance associated with the pncA Gene. This figure presents a detailed analysis of mutations within the pncA gene that are linked to pyrazinamide resistance detected by TBseq® test.

**Table S1** The targeted genes and specific sequences of primers of TBseq® test.

**Table S2** The diagnostic results of each diagnostic method for the enrolled subjects.

**Table S3** Comparison of sensitivity, specificity, PPV, NPV, and AUC among different diagnostic tests among patients clinically TB cases and non-Cases.

**Table S4** The diagnostic accuracy of the MGIT 960 and LJ culture for the diagnosis of tuberculosis.

**Table S5** The diagnostic accuracy of the five tests for the diagnosis of tuberculosis in BALF specimens.

**Table S6** The diagnostic accuracy of the five tests for the diagnosis of tuberculosis in sputum specimens.

**Table S7** Comparison of the accuracy of BALF samples and sputum samples for the diagnosis of tuberculosis.

**Table S8** Proportion of patients with TBseq® test and pDST drug resistance.

**Table S9** The distribution of mutations on drug-resistant patients identified by TBseq® test.

**Table S10** The distribution of co-current mutations across patients detected by TBseq® test.

**Table S11** The discrepancies between TBseq® test and pDST.

**Table S12** Average turnaround time per batch and average cost per sample using TBseq® test

**Table S13** Comparison of the results of the five techniques for the diagnosis of tuberculosis in various sample types.
