# Supplementary material for: Nanopore sequencing for precise detection of Mycobacterium tuberculosis and drug resistance: a retrospective multicenter study in China
Source: J Clin Microbiol. 2025 Mar 19;63(4):e01813-24. doi: 10.1128/jcm.01813-24 (PMC11980377; doi:10.1128/jcm.01813-24)
Supplement: Additional supplemental tables — Tables S5 to S13. [file jcm.01813-24-s0006.docx]

**Table S5 The diagnostic accuracy of the five tests for the diagnosis of** **tuberculosis in BALF specimens**

| **BALF** | **MTB culture**  **( n=558 )** | | **TBseq® test**  **( n=569 )** | | **Xpert MTB/RIF**  **( n=558 )** | | **qPCR**  **( n=492 )** | | **AFB smear**  **( n=541 )** | |
| --- | --- | --- | --- | --- | --- | --- | --- | --- | --- | --- |
| Clinical Diagnosis | Positive | Negative | Positive | Negative | Positive | Negative | Positive | Negative | Positive | Negative |
| Positive  (n=524) | 366 | 148 | 473 | 51 | 396 | 120 | 416 | 57 | 203 | 297 |
| Negative  (n=45) | 3 | 41 | 2 | 43 | 2 | 40 | 4 | 15 | 17 | 24 |
| Sensitivity | 71.2(67.3-75.1) | | 90.3(87.7-92.8) | | 76.7(73.1-80.4) | | 87.9(85.0-90.9) | | 40.6(36.3-44.9) | |
| Specificity | 93.2(85.7-100.0) | | 95.6(89.5-100.0) | | 95.2(88.8-100.0) | | 78.9(60.6-97.3) | | 58.5(43.5-73.6) | |
| PPV | 99.2(98.3-100.1) | | 99.6(99.0-100.2) | | 99.5(98.8-100.2) | | 99.0(98.1-100.0) | | 92.3(88.7-95.8) | |
| NPV | 21.7(15.8-27.6) | | 45.7(35.7-55.8) | | 25.0(18.3-31.7) | | 20.8(11.5-30.2) | | 7.5(4.6-10.4) | |
| AUC | 0.822(0.765-0.879) | | 0.929(0.886-0.972) | | 0.860(0.809-0.910) | | 0.834(0.728-0.941) | | 0.496(0.399-0.593) | |
| PLR | 10.444(3.498-31.178) | | 20.310(5.239-78.743) | | 16.116(4.164-62.377) | | 4.178(1.748-9.985) | | 0.979(0.670-1.430) | |
| NLR | 0.309(0.264-0.362) | | 0.102(0.078-0.133) | | 0.244(0.206-0.290) | | 0.153(0.109-0.214) | | 1.015(0.776-1.326) | |

**Table S6 The diagnostic accuracy of the five tests** **for the diagnosis of tuberculosis in sputum specimens**

| **Sputum** | **MTB culture**  **( n=196 )** | | **TBseq® test**  **( n=200 )** | | **Xpert MTB/RIF**  **( n=197 )** | | **qPCR**  **( n=174)** | | **AFB smear**  **( n=185 )** | |
| --- | --- | --- | --- | --- | --- | --- | --- | --- | --- | --- |
| Clinical Diagnosis | Positive | Negative | Positive | Negative | Positive | Negative | Positive | Negative | Positive | Negative |
| Positive  (n=192) | 148 | 40 | 184 | 8 | 160 | 29 | 164 | 5 | 108 | 70 |
| Negative  (n=8) | 0 | 8 | 0 | 8 | 1 | 7 | 0 | 5 | 2 | 5 |
| Sensitivity | 78.7(72.9-84.6) | | 95.8(93.0-98.7) | | 84.7(79.5-89.8) | | 97.0(94.5-99.6) | | 60.7(53.5-67.9) | |
| Specificity | 100.0(100.0-100.0) | | 100.0(100.0-100.0) | | 87.5(64.6-100.0) | | 100.0(100.0-100.0) | | 71.4(38.0-100.0) | |
| PPV | 100.0(100.0-100.0) | | 100.0(100.0-100.1) | | 99.4(98.2-100.6) | | 100.0(100.0-100.1) | | 98.2(95.7-100.7) | |
| NPV | 16.7(6.1-27.2) | | 50.0(25.5-74.5) | | 19.4(6.5-32.4) | | 50.0(19.0-81.0) | | 6.7(1.0-12.3) | |
| AUC | 0.94(0.864-0.923) | | 0.979(0.007-0.965) | | 0.861(0.721-1.000) | | 0.985(0.972-0.998) | | 0.661(0.457-0.864) | |
| PLR | Inf(NaN-Inf) | | Inf(NaN-Inf) | | 6.772(1.082-42.406) | | Inf(NaN-Inf) | | 3.124(0.654-6.892) | |
| NLR | 0.213(0.162-0.280) | | 0.042(0.021-0.082) | | 0.175(0.115-0.268) | | 0.030(0.012-0.070) | | 0.551(0.333-0.910) | |

**Table S7 Comparison of the accuracy of BALF samples and sputum samples for the diagnosis of tuberculosis.**

|  | **MTB culture** | **TBseq® test** | **Xpert MTB/RIF** | **qPCR** | **AFB smear** |
| --- | --- | --- | --- | --- | --- |
| Sensitivity | P = 0.05803 | P = 0.0247 | P = 0.02962 | P = 0.001027 | P = 5.952e-06 |
| Specificity | P = 1 | P = 1 | P = 0.4143 | P = 0.544 | P = 0.6874 |
| PPV | P = 0.5612 | P = 1 | P = 1 | p= 0.5809 | P = 0.05465 |
| NPV | P = 0.5698 | P = 0.9646 | P = 0.6246 | p= 0.05849 | P = 1 |
| AUC | P = 0.006635 | P = 0.00644 | P = 0.9896 | p= 0.0022 | P = 0.1086 |

**Table S8 Proportion of patients with TBseq® test and pDST drug resistance**

| Drugs | TBseq® test (n1, %) | pDST (n2, %) |
| --- | --- | --- |
| RFP  (n1=720, n2=720) | 212 (29.44) | 195(27.08) |
| INH  (n1=720, n2=689) | 204 (28.33) | 189(27.43) |
| EMB  (n1=720, n2=682) | 99 (13.75) | 71(10.41) |
| Sm  (n1=720, n2=664) | 140 (19.44) | 142(21.39) |
| Lfx  (n1=720, n2=672) | 103 (14.30) | 81 (12.05) |
| Am  (n1=720, n2=664) | 27 (3.75) | 33 (4.97) |
| Cm  (n1=720, n2=663) | 27 (3.75) | 26 (3.92) |

**Table S9 The distribution of mutations on drug-resistant patients identified by TBseq® test.**

| Drug | Gene | Gene mutation (nucleotide position) | Gene mutation  (codon position) | Mutation number | Mutation frequency |
| --- | --- | --- | --- | --- | --- |
| RIF | *rpoB* | 1592C>T | Ser531Leu | 125 | 53.88% |
|  |  | 1532T>C | Leu511Pro | 10 | 4.31% |
|  |  | 1576C>T | His526Tyr | 11 | 4.74% |
|  |  | 1598T>C | Leu533Pro | 10 | 4.31% |
|  |  | 1577A>T | His526Leu | 5 | 2.16% |
|  |  | 1576C>A | His526Asn | 4 | 1.72% |
|  |  | 1576C>G | His526Asp | 4 | 1.72% |
|  |  | 1592C>G | Ser531Trp | 3 | 1.29% |
|  |  | 1546G>T | Asp516Tyr | 3 | 1.29% |
|  |  | 1534A>G | Ser512Gly | 4 | 1.72% |
|  |  | 1547A>T | Asp516Val | 4 | 1.72% |
|  |  | 1547A>G | Asp516Gly | 3 | 1.29% |
|  |  | 1577A>G | His526Arg | 2 | 0.86% |
|  |  | 1455delG | Asn487fs | 3 | 1.29% |
|  |  | 1538A>C | Gln513Pro | 3 | 1.29% |
|  |  | 1270T>G | Phe424Val | 2 | 0.86% |
|  |  | 1508T>C | Phe503Ser | 1 | 0.43% |
|  |  | 1555_1557delAAC | Asn519del | 2 | 0.86% |
|  |  | 1578C>A | His526Gln | 2 | 0.86% |
|  |  | 1349C>T | Ser450Leu | 2 | 0.86% |
|  |  | 1361C>A | Pro454His | 2 | 0.86% |
|  |  | 1532T>G | Leu511Arg | 2 | 0.86% |
|  |  | 1569delG | Leu524fs | 2 | 0.86% |
|  |  | 1577A>G+1576C>G | His526Gly | 2 | 0.86% |
|  |  | 1578C>G | His526Gln | 1 | 0.43% |
|  |  | 1591T>C | Ser531Pro | 1 | 0.43% |
|  |  | 1592C>A | Ser531* | 1 | 0.43% |
|  |  | 1555_1556delAA | Asn519fs | 1 | 0.43% |
|  |  | 1520delG | Gly507fs | 1 | 0.43% |
|  |  | 1522_1523delAC | Thr508fs | 1 | 0.43% |
|  |  | 1522_1527delACCAGC | Thr508_Ser509del | 1 | 0.43% |
|  |  | 1438A>G | Ile480Val | 1 | 0.43% |
|  |  | 1448C>T | Pro483Leu | 1 | 0.43% |
|  |  | 1565C>T | Ser522Leu | 1 | 0.43% |
|  |  | 1547A>C | Asp516Ala | 1 | 0.43% |
|  |  | 1379A>G | Glu460Gly | 1 | 0.43% |
|  |  | 1297_1299 dup TTC | Leu182Ser | 1 | 0.43% |
|  |  | 1360C>T | Pro454Ser | 1 | 0.43% |
|  |  | 1513T>C | Phe505Leu | 1 | 0.43% |
|  |  | 1533dupG | Ser512fs | 1 | 0.43% |
|  |  | 1537C>A | Gln513Lys | 1 | 0.43% |
|  |  | 1540_1542dupTTC | Phe514dup | 1 | 0.43% |
|  |  | 1564T>C | Ser522Pro | 1 | 0.43% |
|  |  | 1577A>C | His526Pro | 1 | 0.43% |
|  |  | 1578_1579delCA | His526fs | 1 | 0.43% |
| INH | *katG* | 944G>C | Ser315Thr | 143 | 67.45% |
|  |  | 944G>A | Ser315Asn | 8 | 3.77% |
|  |  | 1190G>A | Trp397* | 3 | 1.42% |
|  |  | 949A>G | Ile317Val | 2 | 0.94% |
|  |  | 944G>C&945C>A | Ser315Thr | 2 | 0.94% |
|  |  | 890G>T | Gly297Val | 1 | 0.47% |
|  |  | 854G>A | Gly285Asp | 1 | 0.47% |
|  |  | 836G>A | Gly279Asp | 1 | 0.47% |
|  |  | 1180A>G | Thr394Ala | 1 | 0.47% |
|  |  | 1153C>T | Arg385Trp | 1 | 0.47% |
|  |  | 1147T>C | Ser383Pro | 1 | 0.47% |
|  |  | 945C>A | Ser315Arg | 1 | 0.47% |
|  | *inhA* | -15C>T | - | 29 | 13.68% |
|  |  | -8T>C | - | 6 | 2.83% |
|  |  | -8T>A | - | 1 | 0.47% |
|  | *ahpC* | -48G>A | - | 4 | 1.89% |
|  |  | -52C>T | - | 3 | 1.42% |
|  |  | -54C>T | - | 1 | 0.47% |
|  |  | -72C>T | - | 1 | 0.47% |
|  |  | -20C>T | - | 1 | 0.47% |
|  |  | -120_-118delCC | - | 1 | 0.47% |
| EMB | *embB* | 916A>G | Met306Val | 43 | 44.33% |
|  |  | 918G>A | Met306Ile | 21 | 21.65% |
|  |  | 1217G>C | Gly406Ala | 5 | 5.15% |
|  |  | 1139G>A | Ser380Asn | 4 | 4.12% |
|  |  | 1217G>A | Gly406Asp | 3 | 3.09% |
|  |  | 1235C>T | Ser412Leu | 4 | 4.12% |
|  |  | 918G>C | Met306Ile | 4 | 4.12% |
|  |  | 1061A>C | Asp354Ala | 1 | 1.03% |
|  |  | 916A>C | Met306Leu | 2 | 2.06% |
|  |  | 918G>T | Met306Ile | 2 | 2.06% |
|  |  | 953A>G | Asn318Ser | 2 | 2.06% |
|  |  | 1041delT | Leu348fs | 1 | 1.03% |
|  |  | 1216G>A | Gly406Ser | 1 | 1.03% |
|  |  | 932A>G | Asp311Gly | 1 | 1.03% |
|  |  | 956A>C | Tyr319Ser | 1 | 1.03% |
|  |  | 890C>T | Ser297Leu | 1 | 1.03% |
|  |  | 956A>G | Tyr319Cys | 1 | 1.03% |
| Sm | *rpsL* | 128A>G | Lys43Arg | 93 | 70.99% |
|  |  | 263A>G | Lys88Arg | 14 | 10.69% |
|  |  | 125delC | Pro42fs | 2 | 1.53% |
|  |  | 130_132delAAG | Lys44del | 1 | 0.76% |
|  |  | 271C>T | Pro91Ser | 1 | 0.76% |
|  |  | 43_44delAA | Lys15fs | 1 | 0.76% |
|  |  | 123delT | Pro42fs | 1 | 0.76% |
|  |  | 128A>C | Lys43Thr | 1 | 0.76% |
|  |  | 128delA | Lys43fs | 1 | 0.76% |
|  |  | 257G>C | Arg86Pro | 1 | 0.76% |
|  |  | 259G>T | Val87Leu | 1 | 0.76% |
|  |  | 263A>T | Lys88Met | 1 | 0.76% |
|  |  | 26G>A | Arg9His | 2 | 1.53% |
|  |  | 29dupA | Arg12fs | 1 | 0.76% |
|  |  | 307delG | Asp103fs | 1 | 0.76% |
|  |  | 514A>C | - | 1 | 0.76% |
|  | *rrs* | 517C>T | - | 2 | 1.53% |
|  |  | 514A>C | - | 5 | 3.82% |
|  |  | 514A>T | - | 1 | 0.76% |
| Lfx | *gyrA* | 281A>G | Asp94Gly | 37 | 34.91% |
|  |  | 269C>T | Ala90Val | 20 | 18.87% |
|  |  | 281A>C | Asp94Ala | 12 | 11.32% |
|  |  | 271T>C | Ser91Pro | 12 | 11.32% |
|  |  | 280G>A | Asp94Asn | 9 | 8.49% |
|  |  | 280G>T | Asp94Tyr | 4 | 3.77% |
|  |  | 280G>C | Asp94His | 2 | 1.89% |
|  |  | 1381G>A | Asp461Asn | 1 | 0.94% |
|  |  | 1495A>G | Asn499Asp | 1 | 0.94% |
|  |  | 265G>A | Asp89Asn | 1 | 0.94% |
|  |  | 269C>A | Ala90Glu | 1 | 0.94% |
|  | *gyrB* | 1503A>C | Glu501Asp | 3 | 2.83% |
|  |  | 1381G>A | Asp461Asn | 1 | 0.94% |
|  |  | 1684T>C | Phe562Leu | 1 | 0.94% |
|  |  | 1496A>C | Asn499Thr | 1 | 0.94% |
| Am/Cm | *rrs* | 1401A>G | - | 11 | 42.31% |
|  |  | 1452delG | - | 2 | 7.69% |
|  |  | 117_118delAC | - | 1 | 3.85% |
|  |  | 1202delC | - | 1 | 3.85% |
|  |  | 1244delA | - | 1 | 3.85% |
|  |  | 1247delG | - | 1 | 3.85% |
|  |  | 1297delG | - | 1 | 3.85% |
|  |  | 1402C>T | - | 1 | 3.85% |
|  |  | 1474C>A | - | 1 | 3.85% |
|  |  | 290C>T | - | 1 | 3.85% |
|  |  | 302delG | - | 1 | 3.85% |
|  |  | 361delG | - | 1 | 3.85% |
|  |  | 485_486delGA | - | 1 | 3.85% |
|  |  | 1167delG | - | 1 | 3.85% |
|  |  | 1443delC | - | 1 | 3.85% |
| Pyrazinamide | *pncA* | 340A>G | Thr114Ala | 3 | 4.05% |
|  |  | 422A>C | Gln141Pro | 3 | 4.05% |
|  |  | 436G>A | Ala146Thr | 3 | 4.05% |
|  |  | 188A>C | Asp63Ala | 2 | 2.70% |
|  |  | 202T>C | Trp68Arg | 2 | 2.70% |
|  |  | 226A>C | Thr76Pro | 2 | 2.70% |
|  |  | 227C>T | Thr76Ile | 2 | 2.70% |
|  |  | 29A>C | Gln10Pro | 2 | 2.70% |
|  |  | 355T>C | Trp119Arg | 2 | 2.70% |
|  |  | 35A>G | Asp12Gly | 2 | 2.70% |
|  |  | 390_391dupGG | Val131fs | 2 | 2.70% |
|  |  | 545T>C | Leu182Ser | 2 | 2.70% |
|  |  | 134T>G | Val45Gly | 1 | 1.35% |
|  |  | 139A>C | Thr47Pro | 1 | 1.35% |
|  |  | 139A>G | Thr47Ala | 1 | 1.35% |
|  |  | 143A>C | Lys48Thr | 1 | 1.35% |
|  |  | 146A>G | Asp49Gly | 1 | 1.35% |
|  |  | 153C>A | His51Gln | 1 | 1.35% |
|  |  | 169C>G | His57Asp | 1 | 1.35% |
|  |  | 169C>T | His57Tyr | 1 | 1.35% |
|  |  | 19G>A | Val7Ile | 1 | 1.35% |
|  |  | 203G>A | Trp68* | 1 | 1.35% |
|  |  | 203G>C | Trp68Ser | 1 | 1.35% |
|  |  | 20T>G | Val7Gly | 1 | 1.35% |
|  |  | 212A>G | His71Arg | 1 | 1.35% |
|  |  | 215G>A | Cys72Tyr | 1 | 1.35% |
|  |  | 233dupG | Ala79fs | 1 | 1.35% |
|  |  | 233G>A | Gly78Asp | 1 | 1.35% |
|  |  | 269T>G | Ile90Ser | 1 | 1.35% |
|  |  | 286A>G | Lys96Glu | 1 | 1.35% |
|  |  | 289G>A | Gly97Ser | 1 | 1.35% |
|  |  | 289G>T | Gly97Cys | 1 | 1.35% |
|  |  | 28C>T | Gln10* | 1 | 1.35% |
|  |  | 298A>C | Thr100Pro | 1 | 1.35% |
|  |  | 308A>C | Tyr103Ser | 1 | 1.35% |
|  |  | 309C>G | Tyr103* | 1 | 1.35% |
|  |  | 312C>A | Ser104Arg | 1 | 1.35% |
|  |  | 314G>A | Gly105Asp | 1 | 1.35% |
|  |  | 322G>A | Gly108Arg | 1 | 1.35% |
|  |  | 347T>C | Leu116Pro | 1 | 1.35% |
|  |  | 347T>G | Leu116Arg | 1 | 1.35% |
|  |  | 359T>C | Leu120Pro | 1 | 1.35% |
|  |  | 373G>T | Val125Phe | 1 | 1.35% |
|  |  | 386_394delATGTGGTCG | Asp129_Val131del | 1 | 1.35% |
|  |  | 391dupG | Val131fs | 1 | 1.35% |
|  |  | 403A>C | Thr135Pro | 1 | 1.35% |
|  |  | 407A>G | Asp136Gly | 1 | 1.35% |
|  |  | 407delA | Asp136fs | 1 | 1.35% |
|  |  | 407dupA | Asp136fs | 1 | 1.35% |
|  |  | 416T>C | Val139Ala | 1 | 1.35% |
|  |  | 464T>C | Val155Ala | 1 | 1.35% |
|  |  | 476T>G | Leu159Arg | 1 | 1.35% |
|  |  | 490T>C | Ser164Pro | 1 | 1.35% |
|  |  | 503C>T | Thr168Ile | 1 | 1.35% |
|  |  | 515T>C | Leu172Pro | 1 | 1.35% |
|  |  | 525G>T | Met175Ile | 1 | 1.35% |
|  |  | 539T>C | Val180Ala | 1 | 1.35% |
|  |  | 80T>C | Leu27Pro | 1 | 1.35% |
|  |  | 83C>A | Ala28Asp | 1 | 1.35% |

**Table S10** **The distribution of co-current mutations across patients detected by TBseq® test**

| Drug | Patient number | Gene | Gene mutation (nucleotide position) | Gene mutation  (codon position) | pDST |
| --- | --- | --- | --- | --- | --- |
| RIF | P14 | *rpoB* | 1592C>T+1577A>T | Ser531Leu+His526Leu | resistant |
|  | P117 | *rpoB* | 1598T>C+1578C>A | Leu533Pro+ His526Gln | resistant |
|  | P209 | *rpoB* | 1522_1527delACCAGC+1522_1523delAC | Thr508_Ser509del+Thr508fs | susceptible |
|  | P231 | *rpoB* | 1532T>C+ 1270T>G | Leu511Pro+ Phe424Val | susceptible |
|  | P243 | *rpoB* | 1547A>C+1379A>G+1565C>T | Asp516Ala+Glu460Gly+Ser522Leu | resistant |
|  | P311 | *rpoB* | 1592C>T+1508T>C | Ser531Leu+Phe503Ser | resistant |
|  | P409 | *rpoB* | 1546G>T+1532T>C | Asp516Tyr+Leu511Pro | resistant |
|  | P488 | *rpoB* | 1520delG+1532T>C | Gly507fs+Leu511Pro | resistant |
|  | P497 | *rpoB* | 1555_1557delAAC+1555_1556delAA | Asn519del+Asn519fs | resistant |
|  | P498 | *rpoB* | 1576C>A+1270T>G | His526Asn+Phe424Val | resistant |
|  | P536 | *rpoB* | 1591T>C+1592C>A | Ser531Pro+Ser531* | resistant |
|  | P549 | *rpoB* | 1592C>T+1448C>T | Ser531Leu+Pro483Leu | susceptible |
|  | P605 | *rpoB* | 1576C>T+ 1592C>T | His526Tyr+ Ser531Leu | resistant |
|  | P626 | *rpoB* | 1592C>T+ 1438A>G | Ser531Leu+ Ile480Val | resistant |
|  | P668 | *rpoB* | 1598T>C+ 1578C>G | Leu533Pro+ His526Gln | resistant |
|  | P730 | *rpoB* | 1532T>C+ 1578C>A+ | Leu511Pro+ His526Gln | resistant |
|  | P757 | *rpoB* | 1547A>G+ 1532T>C | Asp516Gly+ Leu511Pro | resistant |
| INH | P117 | *katG+katG* | 949A>G+944G>A | Ile317Val+ Ser315Asn | resistant |
|  | P224 | *katG+katG* | 944G>C+854G>A | Ser315Thr+Gly285Asp | susceptible |
|  | P311 | *katG+inhA* | 944G>C+-15C>T | Ser315Thr+- | resistant |
|  | P326 | *katG+inhA* | 1190G>A+-8T>C | Trp397*+- | resistant |
|  | P352 | *katG+katG* | 1147T>C+836G>A | Ser383Pro+Gly279Asp | resistant |
|  | P363 | *katG+ahpC* | 944G>C+-52C>T | Ser315Thr+- | resistant |
|  | P378 | *katG+inhA* | 944G>C+-15C>T | Ser315Asn+- | resistant |
|  | P396 | *inhA+ahpC* | -15C>T+ -48G>A | -+ -+ | resistant |
|  | P511 | *katG+inhA* | 944G>C+-8T>C | Ser315Thr+- | resistant |
|  | P524 | *katG+katG* | 1190G>A+944G>C | Trp397*+Ser315Thr | resistant |
|  | P535 | *katG+katG* | 945C>A+944G>C | Ser315Arg+Ser315Thr | resistant |
|  | P587 | *katG+inhA* | 1153C>T+-15C>T | Arg385Trp+- | susceptible |
|  | P613 | *katG+inhA* | 944G>C+ -8T>C | Ser315Thr+- | resistant |
|  | P650 | *katG+ahpC* | 944G>C+ -48G>A | Ser315Thr+ - | resistant |
|  | P657 | *katG+inhA* | 944G>C+ -15C>T | Ser315Thr+- | resistant |
|  | P754 | *katG+katG* | 949A>G+944G>A | Ile317Val+ Ser315Asn | resistant |
|  | P776 | *katG+ahpC* | 944G>C+ -20C>T | Ser315Thr+- | resistant |
|  | P815 | *katG+katG* | 1180A>G+944G>A | Thr394Ala+ Ser315Asn | resistant |
| EMB | P397 | embB+embB | 916A>G+ 890C>T | Met306Val+ Ser297Leu | resistant |
|  | P626 | embB+embB | 956A>G+ 916A>G | Tyr319Cys+ Met306Val | resistant |
| Sm | P558 | rpsL+rpsL | 26G>A+128A>G | Arg9His+Lys43Arg | resistant |
| Lfx | P118 | *gyrA+gyrA* | 281A>G+ 269C>T | Asp94Gly+ Ala90Val | resistant |
|  | P203 | *gyrA+gyrB* | 271T>C+1503A>C | Ser91Pro+Glu501Asp | susceptible |
|  | P306 | *gyrA+gyrA* | 281A>G+ 280G>A | Asp94Gly+ Asp94Asn | resistant |
|  | P322 | *gyrA+gyrA* | 281A>C+ 269C>T | Asp94Ala+ Ala90Val | susceptible |
|  | P359 | *gyrA+gyrB* | 281A>C+ 1496A>C | Asp94Ala+ Asn499Thr | resistant |
|  | P374 | *gyrA+gyrA* | 269C>T+271T>C | Ala90Val+Ser91Pro | susceptible |
|  | P498 | *gyrA+gyrA* | 269C>T+281A>G | Ala90Val+Asp94Gly | resistant |
|  | P573 | *gyrA+gyrA* | 281A>G+271T>C | Asp94Gly+Ser91Pro | resistant |
|  | P608 | *gyrA+gyrB* | 281A>G+ 1503A>C | Asp94Gly+ Glu501Asp | susceptible |
|  | P647 | *gyrA+gyrA* | 281A>C+ 269C>T | Asp94Ala+ Ala90Val | resistant |
|  | P668 | *gyrA+gyrA+gyrA* | 281A>G+ 269C>T+ 271T>C | Asp94Gly+ Ala90Val+Ser91Pro | resistant |
| Pyrazinamide | P408 | *pncA+pncA* | 355T>C+340A>G | Trp119Arg+Thr114Ala | / |
|  | P513 | *pncA+pncA* | 169C>T+35A>G | His57Tyr+Asp12Gly | / |
|  | P576 | *pncA+pncA* | 28C>T+ 203G>A | Gln10*+ Trp68* | / |

**Table S11 The discrepancies between TBseq® test and pDST**

|  |  | RFP | INH | EMB | Sm | Am | Cm | Lfx |
| --- | --- | --- | --- | --- | --- | --- | --- | --- |
| pDST(-) | TBseq® test(+) | 23 | 23 | 37 | 10 | 2 | 8 | 41 |
| pDST(+) | TBseq® test(-) | 1 | 10 | 1 | 6 | 0 | 1 | 5 |
| pDST(-) | TBseq® test  (Not_TB) | 48 | 41 | 47 | 38 | 38 | 39 | 38 |
| pDST(+) | TTBseq® test  (Not_TB) | 5 | 7 | 1 | 8 | 9 | 7 | 8 |

**Table S12** **Average turnaround time per batch and average cost per sample using TBseq® test**

| **No.** | **Steps in the sequencing-based workflow** | **Mean Turnaround time per batch**  **(hour)** | **SD** | **Mean Cost per sample**  **(USD)** | **SD** |
| --- | --- | --- | --- | --- | --- |
| 1 | DNA extraction | 1 | 0.1891 | 3.2 | 0.2317 |
| 2 | Target enrichment  (multiplex PCR) | 4 | 0.1267 | 3 | 0.2961 |
| 3 | Library preparation | 2 | 0.1213 | 7.5 | 0.4801 |
| 4 | Quality control | 0.5 | 0.1711 | 2 | 0.2732 |
| 5 | Sequencing | 7 | 0.4056 | 40 | 1.1623 |
| 6 | Generating reports | 1.5 | 0.1904 | 0 | 0 |
| 7 | Total turnaround time | 16 | 0.8113 | 55.7 | 1.9407 |

**Table S13 Comparison of the results of the five techniques** **for the diagnosis of tuberculosis in various sample types**

|  |  | **MTB culture** | | | **TBseq® test** | | | **Xpert MTB/RIF** | | | | **qPCR** | | | | | | **AFB smear** | | | | |
| --- | --- | --- | --- | --- | --- | --- | --- | --- | --- | --- | --- | --- | --- | --- | --- | --- | --- | --- | --- | --- | --- | --- |
| **Sample type** | Clinical Diagnosis | Positive | | Negative | Positive | Negative | | Positive | Negative | | | Positive | | Negative | | | | Positive | | Negative | | |
| **Pus** | Positive  (n=14) | 7 | | 5 | 13 | 1 | | 11 | 3 | | | 10 | | 1 | | | | 3 | | 9 | | |
|  | Negative  (n=1) | 0 | | 1 | 0 | 1 | | 0 | 1 | | | 0 | | 0 | | | | 0 | | 1 | | |
| **Pleural effusion and Ascites** | Positive  (n=4) | 2 | | 2 | 4 | 0 | | 2 | 2 | | | 2 | | 0 | | | | 0 | | 4 | | |
|  | Negative  (n=1) | 1 | | 0 | 1 | 0 | | 1 | 0 | | | 1 | | 0 | | | | 0 | | 1 | | |
| **Urine** | Positive  (n=5) | 5 | 0 | | 5 | 0 | | 4 | 1 | | | | 5 | | 0 | | | | 1 | 4 | | |
|  | Negative  (n=0) | 0 | 0 | | 0 | 0 | | 0 | 0 | | | | 0 | | 0 | | | | 0 | 0 | | |
| **Tissue** | Positive  (n=19) | 7 | 12 | | 16 | 3 | | 13 | | 4 | | 14 | | | | 5 | | 3 | | | 13 | |
|  | Negative  (n=2) | 0 | 2 | | 1 | 1 | | 0 | | 1 | | 1 | | | | 1 | | 0 | | | 1 | |
| **CSF** | Positive  (n=14) | 4 | | 10 | 7 | | 7 | 3 | | | 8 | 7 | | | | | 7 | 2 | | | | 1 |
|  | Negative  (n=0) | 0 | | 0 | 0 | | 0 | 0 | | | 0 | 0 | | | | | 0 | 0 | | | | 0 |
